# Systematic Surveys of Iron Homeostasis Mechanisms Reveal Ferritin Superfamily and Nucleotide Surveillance Regulation to be Modified by PINK1 Absence

**DOI:** 10.3390/cells9102229

**Published:** 2020-10-02

**Authors:** Jana Key, Nesli Ece Sen, Aleksandar Arsović, Stella Krämer, Robert Hülse, Natasha Nadeem Khan, David Meierhofer, Suzana Gispert, Gabriele Koepf, Georg Auburger

**Affiliations:** 1 Experimental Neurology, Medical School, Goethe University, 60590 Frankfurt am Main, Germany; key@stud.uni-frankfurt.de (J.K.); nesliecesen@gmail.com (N.E.S.); arsovicalexandar@gmail.com (A.A.); stella.kraemer@yahoo.de (S.K.); robert.huelse@gmail.com (R.H.); natzkhan.203@gmail.com (N.N.K.); Gispert-Sanchez@em.uni-frankfurt.de (S.G.); Gabriele.Koepf@kgu.de (G.K.); 2 Faculty of Biosciences, Goethe-University, Altenhöferallee 1, 60438 Frankfurt am Main, Germany; 3 Max Planck Institute for Molecular Genetics, Ihnestraße 63-73, 14195 Berlin, Germany; Meierhof@molgen.mpg.de

**Keywords:** synuclein, CPT1A, MMP14, PYGL, *Tfrc*, *Ireb2*, *Pgrmc1*, *Hmox1*, *Cyp46a1*, *Slc11a2*, *Slc25a37*, iron overload versus deprivation, nucleotide metabolism, neurodegeneration

## Abstract

Iron deprivation activates mitophagy and extends lifespan in nematodes. In patients suffering from Parkinson’s disease (PD), PINK1-PRKN mutations via deficient mitophagy trigger iron accumulation and reduce lifespan. To evaluate molecular effects of iron chelator drugs as a potential PD therapy, we assessed fibroblasts by global proteome profiles and targeted transcript analyses. In mouse cells, iron shortage decreased protein abundance for iron-binding nucleotide metabolism enzymes (prominently XDH and ferritin homolog RRM2). It also decreased the expression of factors with a role for nucleotide surveillance, which associate with iron-sulfur-clusters (ISC), and are important for growth and survival. This widespread effect included prominently *Nthl1-Ppat-Bdh2*, but also mitochondrial *Glrx5*-*Nfu1*-*Bola1*, cytosolic *Aco1-Abce1-Tyw5*, and nuclear *Dna2*-*Elp3*-*Pold1*-*Prim2*. Incidentally, upregulated *Pink1*-*Prkn* levels explained mitophagy induction, the downregulated expression of *Slc25a28* suggested it to function in iron export. The impact of PINK1 mutations in mouse and patient cells was pronounced only after iron overload, causing hyperreactive expression of ribosomal surveillance factor *Abce1* and of ferritin, despite ferritin translation being repressed by IRP1. This misregulation might be explained by the deficiency of the ISC-biogenesis factor GLRX5. Our systematic survey suggests mitochondrial ISC-biogenesis and post-transcriptional iron regulation to be important in the decision, whether organisms undergo PD pathogenesis or healthy aging.

## 1. Introduction

Intracellular iron metabolism in mammalian cells is crucial for their proper functions. Free iron molecules can be toxic to cells, leading to the production of ROS (reactive oxygen species) and lipid peroxidation terminating in a programmed cell death known as ferroptosis [1], so there have to be strict regulatory mechanisms. Iron metabolism affects the whole cell, since iron is taken up from extracellular space either via transferrin or in a transferrin-independent manner, then reduced for detoxification and distributed within cells, for example to mitochondria, where iron is utilized for iron-sulfur-cluster (ISC) production and heme generation [2,3,4]. The correct function of dozens of proteins in mitochondria, cytosol, and nucleus depends on the insertion of ISC [5,6], so the regulation of iron homeostasis is crucial. ISC are unstable during oxidative stress periods, but play important roles in a wide range of cellular reactions, e.g., electron transfer, catalysis of enzymatic reactions, regulation of gene expression (e.g., via association with IRP1/IRP2 (iron regulatory proteins) to exert post-transcriptional control over ferritin and transferrin receptor levels), and in the quality control of nucleotides, thus controlling genome integrity [7,8,9]. Similarly, heme is needed as a cofactor of cytochrome proteins within mitochondria, but also of cytosolic cytochrome P450 proteins, globins, iron-regulatory proteins, peroxidases, catalase, and specific ion-channels [10].

Recently, it was shown in the nematode *Caenorhabditis elegans* that an extrinsic factor, namely the availability of iron, has a strong impact on lifespan. The suppression of iron uptake by a chelator drug, as well as the silencing of *Fxn* (FRATAXIN) (cursive lowercase letters refer to the DNA/RNA level in rodents, while uppercase letters refer to the protein) as a mitochondrial ISC biogenesis factor, both extended the lifespan via mitochondrial stress and activation of PINK1/PARKIN-dependent mitophagy. Downstream effects of this pathological scenario included the elevated expression of globins, which bind to iron in the form of heme [11]. It was also reported that natural inducers of mitophagy, such as urolithin A, can extend the lifespan in *C. elegans* [12]. We were intrigued by these observations since a converse situation is observed in man: Defective mitophagy due to *Pink1/Prkn* mutations shortens the lifespan and leads to the accumulation of iron during a neurodegenerative process that we know as Parkinson’s disease (PD) [13,14,15].

The serine-threonine kinase PINK1 (PTEN induced kinase 1) associates with the outer mitochondrial membrane and phosphorylates cytosolic proteins to coordinate the PARKIN-dependent autophagic degradation of damaged or aged mitochondria, in a process known as mitophagy [16,17,18]. *PINK1* and *PRKN* (encoding PARKIN) get transcriptionally induced in human neuroblastoma cells after serum deprivation or nutrient starvation [19], linking dietary restriction to mitophagy. Mutations in *PINK1* and *PRKN* lead to autosomal recessive juvenile-onset variants of PD, which were named PARK6 and PARK2, respectively [14,20]. Iron distribution is altered in the brains of all PD patients [21,22], with a preferential increase of iron levels in the midbrain substantia nigra [23,24], where the loss of dopaminergic neurons is observed. These findings add to the established concept that iron accumulation contributes to neurodegenerative processes. In dopaminergic midbrain neurons, much of the stored iron is absorbed onto neuromelanin granules, while other neurons and brain glial cells can only deposit it as ferritin protein complexes [25]. It remains unclear to what degree in diverse cells the pathological redistribution of excess iron occurs towards the labile iron pool (LIP), to mitochondria, or to ferritin with its ferroxidase site, where ferrous iron (+2 oxidation state) can be converted to ferric iron (+3 oxidation state) and thus stored [26]. It is conceivable that altered turnover of iron-containing proteins contributes to the iron toxicity in PD. One piece of evidence was found in a neurotoxic PD model via 5-day acute exposure to the respiratory complex-I inhibitor 1-methyl-4-phenyl-1,2,3,6-tetrahydropyridine (MPTP), where an acute increase of ferritin light-chain and mitochondrial ferritin (*Ftmt*) was reported together with a protective function of mitochondrial ferritin overexpression [27].

Detailed studies of PD pathogenesis confirmed that (i) *PINK1* mutations trigger iron accumulation in the midbrain of patients [28], (ii) *Pink1* deficiency-mediated iron accumulation may involve degradation of mitochondrial membrane iron transporters SLC25A37 (*Mfrn1*) and SLC25A28 (*Mfrn2*) (solute carrier family 25, member 37/28) [29,30], (iii) iron chelation-induced mitophagy can be observed in PARK2 patient primary fibroblasts [31], (iv) *Pink1*-dependent phenotypes in flies can be suppressed by mitochondrial aconitase (ACO2), while superoxide-dependent inactivation of the ACO2 [4Fe-4S] cluster triggers iron toxicity that is reversed by mitochondrial ferritin overexpression [32]. Thus, there is a close link between the mitochondrial dysfunction underlying PD on the one hand, with the homeostasis of iron, ISC, and heme on the other hand. It is important now to elucidate the relevant molecular events of iron homeostasis and how it is modulated in PD, to define molecular biomarkers of PD progression, and to understand how extrinsic factors may modify the disease course.

In the chronic state of mutant brain tissue, it is very hard to detect these anomalies. Mitophagy is relevant for only a few among hundreds or thousands of mitochondria per cell at any given time, and the accumulation of iron is an insidious process over decades in PD patient brain, so the compensatory efforts needed are minimal. Therefore, global expression profiles of *Pink1*^−/−^ mouse brain showed only subtle evidence of deficient mitophagy and altered mitochondrial biogenesis [20,33,34,35], the dysregulated expression of heme-related transcripts *Hmox1* (Heme oxygenase 1) and *Hebp1* (Heme binding protein 1) was noted only upon culture of mouse *Pink1*^−/−^ primary cortical neurons [35], and limited survival of the *Pink1*^−/−^ mouse was observed only after additional overexpression of toxic alpha-synuclein (SNCA) [20,36]. In general, PINK1- and PARKIN-deficient mice show signs of altered mitophagy and neurodegeneration only in the presence of further stressors such as mitochondrial mutations, exhaustive exercise, or bacterial infections [37,38,39,40]. A lifespan effect was also not detectable in *C. elegans Pink1*- and *Parkin*-mutants. In contrast, the survival of *Drosophila melanogaster* flies with depletion of PINK1 or PARKIN was significantly shortened by degeneration of wing muscles, due to the massive exercise and energetic demand during flight [20,41,42,43].

Not only altered mitophagy but also autophagy and mitochondrial dysfunction in general, have strong effects on iron homeostasis and lifespan, as was demonstrated in *C. elegans* for the so-called mit-mutants, where dysfunctions of the electron transfer chain trigger unexpected longevity [44,45,46]. In another well-established model of rapid aging, the fungus *Podospora anserina*, a simple depletion of the mitochondrial matrix protease ClpP (Caseinolytic mitochondrial matrix peptidase proteolytic subunit) results in a prolonged lifespan [47]. Again, mice with *ClpP* deletion were reported to have altered survival with higher resistance to metabolic stress and bacterial infections [48,49], as well as iron and hemoglobin accumulation (https://www.mousephenotype.org/data/genes/MGI:1858213). In contrast to *Pink1* mutant cells [17,50], stable *ClpP* mutants showed no evidence of oxidative stress [51], enhancing the doubts whether ROS have a central process in the control of lifespan [52,53] and emphasizing the notion that iron levels may be more relevant stressors than ROS.

Although our studies now were done in vitro with peripheral cells using massive acute iron stress and are thus limited to extreme situations never observed *in vivo*, we believe that similar subtle long term effects can occur in the nervous system and might contribute to neurodegenerative diseases.

## 2. Results

### 2.1. Global Proteome Profile Adaptations upon Iron Overload and Iron Depletion in WT and Pink1^−/−^ Mouse Embryonic Fibroblast (MEF) Cells

**Figure 1:**
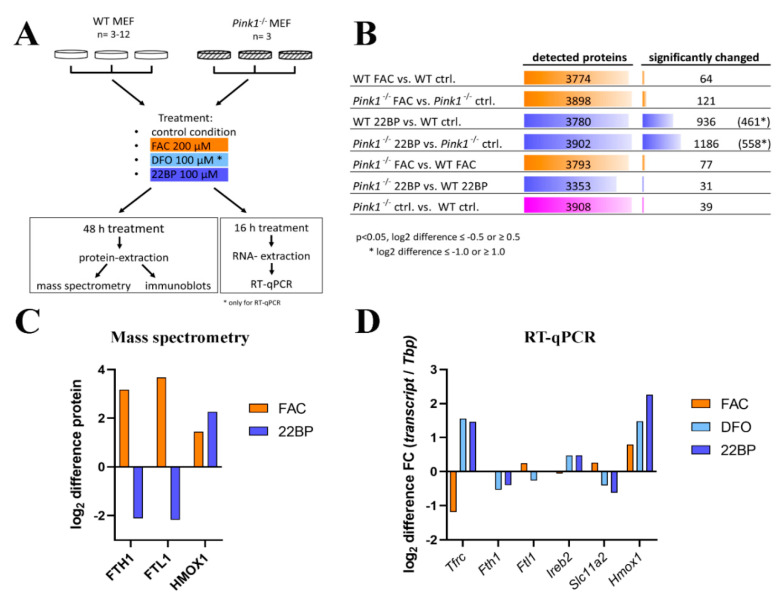
(**A**) Schematic representation of the experimental setup. Wildtype (WT) and *Pink1*^−/−^ mouse embryonic fibroblast (MEF) were either left untreated or incubated with ferric ammonium acid (FAC), DFO (Deferoxamine), and 2,2′-Bipyridine (22BP). mRNA expression was analyzed by RT-qPCR after 16 h incubation, while protein abundances were analyzed by mass spectrometry and quantitative immunoblots after 48 h incubation. The FAC treatment is highlighted in orange, whereas the two iron chelator treatments are highlighted in light and dark blue, as the color code for the entire manuscript. (**B**) Summary of mass spectrometry results for the seven conditions studied in comparison, showing the total number of detected proteins and the number of factors with significantly changed abundance, with the respective cutoff values for the significance and the fold-change (shown as log_2_ difference). The analysis of WT 22BP versus WT ctrl. and of *Pink1*^−/−^ 22BP versus *Pink1*^−/−^ ctrl. revealed so many significant factors that a log_2_ difference of 1.0 was used as the cutoff for downstream pathway enrichment analyses. (**C**) As measures of quality control for the culture incubations, the fold-changes as log_2_ differences are shown for well-established iron homeostasis factors, as detected in mass spectrometry, in comparison to (**D**) the respective log_2_ differences by RT-qPCR for such key iron homeostasis genes. The RT-qPCR results were normalized to *Tbp* expression levels (Tata-binding protein encoding mRNA). ctrl. = untreated control condition.

The initial quality evaluation of stressed WT MEF (wildtype mouse embryonic fibroblasts) cultures showed that iron overload mediated by ferric ammonium citrate (FAC) exposure triggered relatively few abundance changes, in comparison to iron shortage mediated by 2,2′-Bipyridine (22BP) administration, and *Pink1*-ablation also caused few effects (Figure 1B and Supplementary Table S1), upon mass spectrometry and subsequent statistical analysis. Venn diagram analyses were employed to identify factors that were dysregulated in more than one condition (Supplementary Table S1).

Normal culture media contain iron concentrations that were maximized to stimulate cell growth, so iron depletion is expected to have the highest impact. Further plausibility assessments showed that the heavy and light ferritin chain subunits (FTH1 and FTL1) were in direct correlation to iron availability; also the heme oxygenase (HMOX1) increased after FAC treatment (Figure 1C), confirming well-known regulations. A strong induction of ferritin protein by FAC administration over 24 h, for example, was previously documented in rat primary neurons by a mass spectrometry profiling study [54]. Our additional tests by RT-qPCR also for less abundant factors demonstrated the known indirect correlation of iron availability with the transferrin receptor (encoded by the gene *Tfrc*) and aconitase-3 (*Ireb2*) levels, as well as its direct correlation with the divalent metal transporter 1 (*Slc11a2,* Solute carrier family 11 member 2) mRNA levels (Figure 1D). Taken together, the culture settings used validated previous knowledge and provided a useful approach for systematic explorations into yet unknown effects.

#### 2.1.1. FAC-Effects

**Figure 2:**
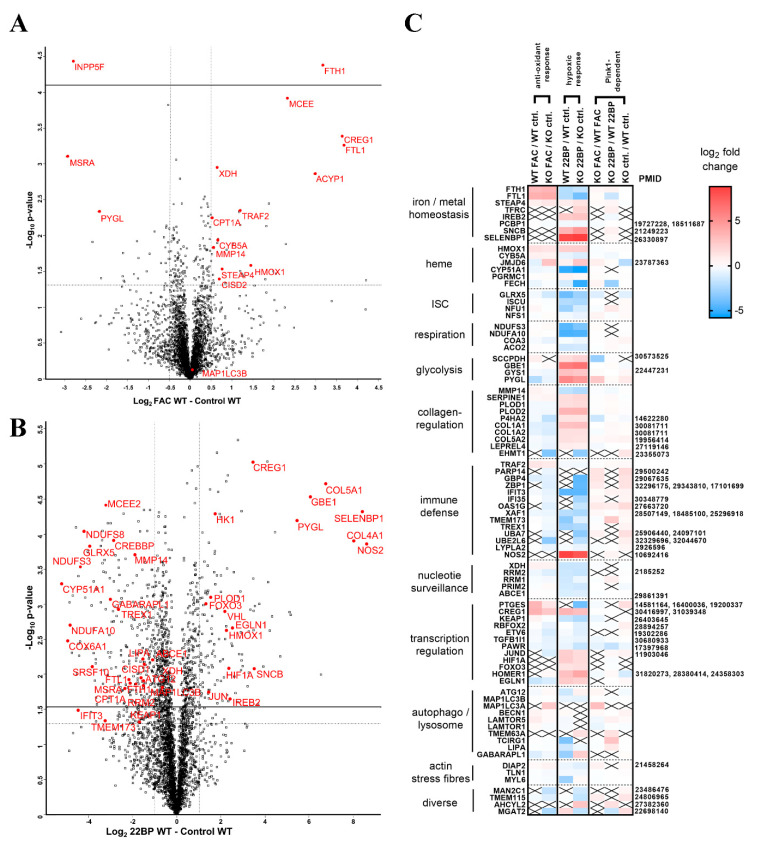
Global proteome profiles presented as Volcano plots, where significance (−log_10_ *p*-value) is shown on the Y-axis (actual threshold visualized by a solid line (FDR = 0.05), nominal threshold by a dotted line, *p* = 0.05), while fold-changes (log_2_ differences) are presented on the X-axis (dotted line refers to cutoff used for subsequent Search Tool for the Retrieval of Interacting Genes/Proteins (STRING) interaction and pathway enrichment analyses). Gene symbols were used to identify relevant proteins. Iron overload effects via FAC administration are illustrated in (**A**), iron depletion effects by 22BP administration in (**B**). The heat map in (**C**) summarizes the fold changes of proteins that were repeatedly dysregulated with nominal significance upon mass spectrometry, together with their categorization in pathways on the left margin, and relevant literature as PubMed database of medical literature, reference IDentifier number (PMID) on the right. Log_2_ fold changes of abundance are shown in blue (negative) or red (positive), x represents non-detection of the factor. ctrl. = untreated control condition.

The comparison of WT MEF cells, FAC treated versus untreated, revealed an upregulation for the ferritin chain heavy subunit FTH1 and downregulation for the transferrin-receptor regulator INPP5F (Inositol Polyphosphate-5-Phosphatase F) (also known as SAC2), with additional factors showing nominal significance (see Table 1 and Figure 2A).

Importantly, several factors with dysregulations after FAC treatment showed converse regulation after 22BP treatment, namely iron-binding XDH (Xanthine dehydrogenase), mitochondrial transporter CPT1A (Carnitine palmitoyltransferase 1A), collagen-degrading MMP14 (Matrix metallopeptidase 14), and glycogen-mobilizing PYGL (Glycogen phosphorylase L). Heat maps were generated to visualize how strongly and in which direction such factors changed after FAC versus 22BP administration, and how this was modulated by *Pink1*-genotype, ordered by the pathways involved and highlighting the novel impact of iron deficiency on nucleotide synthesis/surveillance factors (Figure 2C).

Overall, statistical analyses of all these factors for the enrichment of interactions, Gene Ontology (GO)-terms, pathways, and protein domains were performed with the STRING webserver [55]. This revealed multiple molecules involved in iron-binding, mitochondria, vesicles, and autophagy to show increased abundance with significant enrichment after FAC, while factors in glycolysis and metabolic pathways consistently showed decreased abundance after FAC (Supplementary Figure S1).

The comparison of *Pink1*^−/−^ MEF cells, FAC treated versus untreated, showed higher responsiveness (Figure 1B and Figure 2C). Among so many new findings, we focused on factors with a key role for iron homeostasis, or for the mechanism of iron involvement in PD, as well as factors that are ISC- or heme-associated (Table 1, Supplementary Figure S2A). The iron-regulatory protein 2 (IRP2 aka IREB2) was not detectable after FAC treatment or in untreated condition, in WT or *Pink1*^−/−^ cells, while it was significantly elevated after 22BP exposure. A significant upregulation of CISD2 (CDGSH iron-sulfur domain-containing protein 2) in WT cells (Figure 2A) is noteworthy in the context of post-transcriptional regulation of iron homeostasis, given the role of its homologs for the ISC regeneration in IRP1 [56,57,58,59].

It is important to note that several of these inductions are part of the known antioxidant ferroptosis-mitigating response under the control of the transcription factor NRF2 (Nuclear factor, erythroid 2 like 2, encoded by the *Nfe2l2* gene), with experimental confirmation for FTL1/FTH1/HMOX1/NQO1 [NAD(P)H quinone dehydrogenase 1] [60,61]. According to GeneCards database (accessed on 31 July 2020), the NRF2 binding site exists also in the promoter of genes coding for CYB5A (Cytochrome B5 type A)/CISD2/MCEE (Methylmalonyl-CoA Epimerase)/CREG1 (Cellular repressor of E1A stimulated genes 1)/TRAF2 (TNF receptor-associated factor 2)/ACYP1 (Acylphosophatase 1)/NSMCE2 (Non-structural maintenance of chromosomes element 2 homolog)/MAP1LC3A (Microtubule-associated protein 1 light chain 3 alpha)/GPR126 (G-protein coupled receptor 126)/PTGES (Prostaglandin E synthase)/SERPINE2 (Serpin family E member 2), whereas STEAP4 (Six transmembrane epithelial antigen of the prostate)/JMJD6 (Jumonji domain protein 6)/ARMC1 (Armadillo repeat containing 1)/GPSM2 (G Protein signaling modulator 2)/MSRA (Methionine sulfoxide reductase A) promoters contain only the ARNT binding site (Aryl hydrocarbon receptor nuclear translocator) needed by the transcription factor HIF1A (Hypoxia-Inducible Factor 1-alpha) to control hypoxia responses. Indeed, among the many effects triggered by 22BP treatment in *Pink1*^−/−^ cells (Supplementary Figure S2B), dysregulations that just reached nominal significance were detected for HIF1A and the NRF2 regulator KEAP1 (Kelch-like ECH associated protein 1), in opposite direction.

The reproducibility of dysregulations between WT cells and *Pink1*^−/−^ cells was assessed, and all consistent adaptations both to FAC and to 22BP were studied regarding enrichment among them for interactions, GO-terms, pathways, and protein domains, using the STRING webserver (Supplementary Figure S3). Apart from the central adaptation of iron homeostasis, ISC biogenesis, and transcription factors, this approach confirmed the impact on mitochondria, heme-containing globins, glycolysis, autophagy, and collagen. In addition, iron shortage triggered a decreased abundance of nucleotide synthesis and surveillance factors, a pathway effect that was not appreciated in previous work.

#### 2.1.2. 22BP-Effects

Upon comparison of WT MEF cells, 22BP-mediated iron depletion triggered numerous abundance changes (see Supplementary Table S1, Table 2, Figure 2B,C). As mentioned above, FTH1-FTL1, CPT1A, MMP14, XDH, and PYGL showed converse regulation after 22BP versus FAC, whereas HMOX1 and CREG1 were upregulated in both conditions.

Downregulations with probable relevance to PINK1 functions among the iron-binding proteins were observed for the iron-sulfur-complex biosynthesis factor GLRX5 (Glutaredoxin 5) and the iron-sulfur-complex regeneration factor CISD1 that repairs damaged IRP1. Downregulations occurred also for numerous ISC-containing factors, e.g., in the respiratory chain, as well as heme-associated proteins. Other downregulations were found for the serine and arginine-rich splicing factor SRSF10 and the toxic nucleotide sensor IFIT3 (Interferon-induced protein with tetratricopeptide repeats 3) that were implicated in PINK1-associated PD [35]. Among the factors implicated in PD pathogenesis, upregulations were found for FOXO3 (Forkhead box O3) as the mediator of toxicity of the ferrireductase alpha-synuclein (SNCA) toxicity, and for beta-synuclein (SNCB) as an antagonist of SNCA [62,63,64,65,66].

Statistical enrichments among all nominally dysregulated factors were identified with the STRING webserver (accessed on 16 April 2020). Among the upregulations (Supplementary Figure S4A), the hypoxia pathway, apoptosis, glucose metabolism, and collagen formation were prominently affected. Among the downregulations (Supplementary Figure S4B), the mitochondrial respiratory chain, nuclear factors, rRNA processing, and autophagosome pathways stood out. In both directions, iron response and binding factors were enriched.

Upon comparison of *Pink1*^−/−^ cells MEF cells, 22BP treatment affected a myriad of factors (Figure 1B, Supplementary Figure S2B), emphasizing the strong impact of iron chelator therapy on this cell model of PD. Although the analysis of our dataset in MEF cells is important to explore the benefits and potential adverse effects of such a treatment, an exhaustive evaluation is impossible until future proteome profiles of neural cells are also available. Upon STRING enrichment analysis, changes of metabolism and ribosome/RNA factors were prominent among KEGG/Reactome pathways, with false discovery rates of 2e-36 and 4e-27, respectively.

Filtering those factors that responded consistently to 22BP, both in WT and also in *Pink1*^−/−^ cells, are highlighted in Supplementary Figure S3, as e.g., GLRX5, CISD1, FOXO3, and SNCB, again with downstream effects on ISC-containing factors, e.g., in the respiratory chain, as well as heme-associated proteins. As already mentioned, a deficient abundance of several iron-binding nucleotide synthesis and RNA/DNA quality control factors appeared there as new conspicuous findings.

#### 2.1.3. Pink1^−/−^ Effects

To understand the impact of a Parkinson-triggering PINK1 loss-of-function mutation among these regulations, the analogous experiments with FAC/22BP administration were performed in comparisons between *Pink1*^−/−^ versus WT MEF cells (Supplementary Table S1, Table 3).

After iron overload with FAC (Supplementary Figure S2C), the *Pink1*^−/−^ cells showed upregulations of MAP1LC3A probably as an effort to promote Parkin-independent mitophagy [67], and of SLC25A11 (Solute carrier family 25 member 11)/PANK4 (Pantothenate kinase 4)/PYGL/NUCKS (Nuclear casein kinase and cyclin dependent kinase substrate 1) as evidence for excessive mitochondrial metabolic performance, as well as innate immune responses such as SPP1 (Secreted phosphoprotein 1 possibly due to mitochondrial accumulation in this PD variant [68,69,70]. Downregulations occurred for the myosin light chain MYL6 as a putative interactor of the iron chaperone PCBP1 (Poly(RC) binding protein 1), as well as several mitochondrial and lysosomal factors, while the affection of ribosomal and spliceosomal factors like RPF2 (Ribosome production factor 2 homolog) and BUD31 (BUD31 homolog) stood out as novel insights (Table 3).

After iron depletion with 22BP (Supplementary Figure S2D), the *Pink1*^−/−^ cells showed notable downregulations of the autophagy factor ATG4B (Autophagy related 4B cysteine peptidase), responsible for cleavage of MAP1LC3A and its homologs, as well as dysregulations of 3 heme-associated factors (ferrochelatase (FECH), HMOX1, cytochrome B5 type A (CYB5A)). Relevant upregulations were detected for the cAMP-regulated DNA-damage sensor and mitosis initiator ARPP19 (cAMP regulated phosphoprotein 19), the splicing factor SRSF10 that was previously identified as PINK1-regulated [35], the circadian transcriptional coactivator CREBBP (CREB binding protein) that was implicated in PD [71], and the DNA-repair factor RRM1 (Ribonucleotide reductase regulatory subunit) that interacts with ferritin superfamily member RRM2 (Table 3).

During untreated culture conditions, the *Pink1*^−/−^ cells exhibited an upregulation (Supplementary Figure S2E) for the autophagy factor BECN1 (Beclin 1) in a probable effort to facilitate Parkin-dependent mitophagy, together with downregulation for the ubiquitin-like autophagy factor ATG12 (Autophagy related 12). Other downregulations were notable for the iron-sulfur-complex biogenesis factor GLRX5, the iron-binding histone modulator and *FECH*-splicing factor JMJD6, and the ferritin interactor PCBP3, suggesting a chronic PINK1 impact on ISC/heme biogenesis and LIP even in conditions without stress. Further downregulations occurred for the PARK7-homologous peptidase PDDC1 (Glutamine amidotransferase-like class 1 domain containing 1; which interacts with the ferroptosis-induced CREB-associated PRKN-regulator TRIB3 (Tribbles pseudokinase 3)), and for the calcium-excitation factor HOMER1 (Homer scaffold protein 1) that was implicated in PD.

Overall, these data tentatively identify *Pink1*^−/−^ effects on 8 iron/ISC/heme homeostasis factors (TCIRG1 (T Cell immune regulator 1), MYL6, PCBP3, GLRX5, FECH, JMJD6, CYB5A, GYPC (Glycophorin C)), 8 unspecific mitochondrial factors (SLC25A11, SCCPDH (Saccharopine dehydrogenase), HMGCL (Mitochondrial 3-Hydroxy-3-Methylglutaryl-CoA Lyase), PMPCA (Peptidase, mitochondrial processing subunit alpha), MRPS35 (Mitochondrial ribosomal protein S35, MRPS36 (Mitochondrial ribosomal protein S36), CCDC58 (Coiled-coil domain containing 58), ACSL3 (Acyl-CoA synthetase long-chain family member 3)), 6 autophagy factors (MAP1LC3A, ATG4B, ATG12, ATG24A, BECN1, NUP160 (Nucleoporin 160)), 5 lysosomal factors (LAMTOR1 (Late endosomal/lysosomal adaptor 1), LIPA (Lipase A) TMEM63A (Transmembrane protein 63A), LAMTOR5 (Late endosomal/lysosomal adaptor 5), TCIRG1), 5 RNA splicing and surveillance modulators (BUD31, SRSF10, EXOSC4, EXOSC10 (Exosome component 4/10), RBFOX2 (RNA binding fox-1 homolog 2)), 5 DNA-associated/-repair factors (NUCKS, ARPP19, CREBBP, RRM1, UPP1 (Uridine phosphorylase 1)), and 4 innate immunity factors (MYD88 (Myeloid differentiation primary response protein MyD88), IRF2BP1 (Interferon regulatory factor 2 binding protein 1), SPP1, AMDHD2 (Amidohydrolase domain containing 2)). This list is in good agreement with the known functions of PINK1 in mitochondrial degradation via the autophago-lysosomal pathway and with the iron accumulation in PINK1-mutant brain. There was no *Pink1*-dependent factor with converse regulation during iron excess versus iron shortage.

### 2.2. Transcriptional Analyses of Cellular Iron Homeostasis Factors in WT MEF in Response to Altered Iron Levels

**Figure 3:**
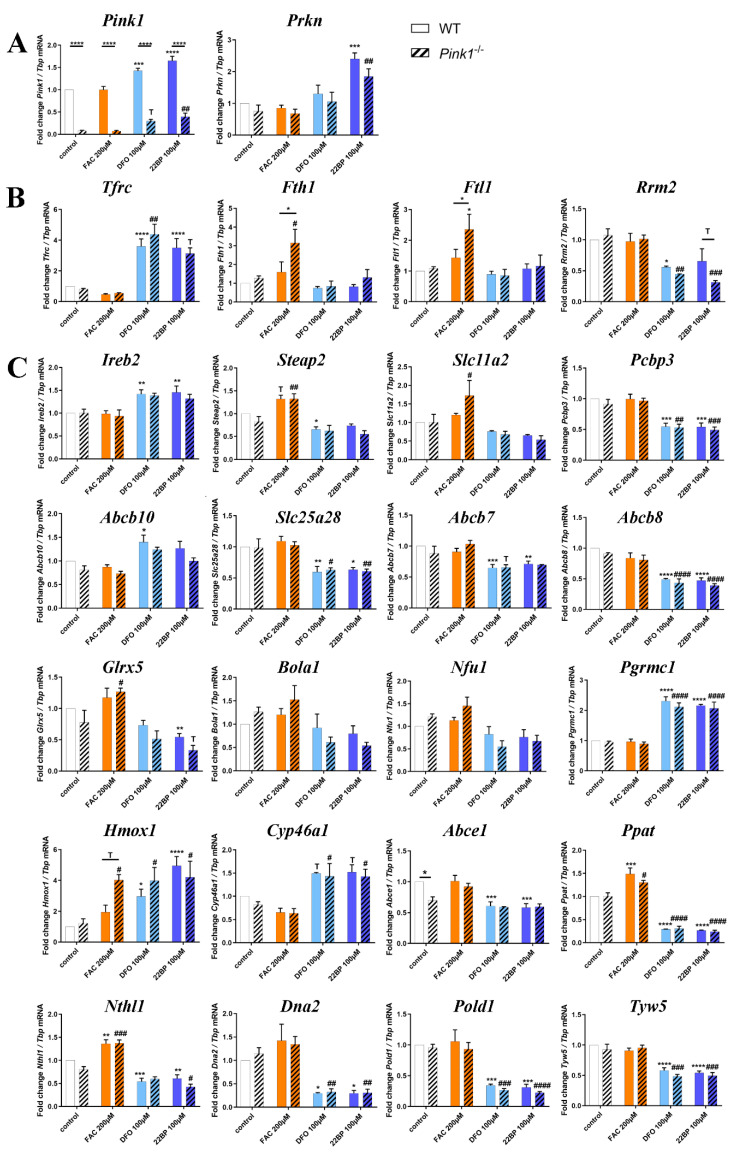
Synopsis of mRNA expression dysregulations upon RT-qPCR in MEF cells (*n* = 3 WT for *Pink1* and *Prkn*, else 6–12 WT versus 3 *Pink1*^−/−^ for all other factors) after iron manipulation, focusing on (**A**) the mitophagy modulators *Pink1* and *Prkn*, (**B**) key modulators of cellular iron uptake, storage, export, (**C**) iron transport and processing, mitochondrial iron homeostasis, heme production/turnover, ISC-biogenesis, and ISC-binding. All factors in (**B**,**C**) are presented in their approximate order of action during cellular iron homeostasis. Their expression adaptation was documented after iron overload (FAC) and under two different iron depletion conditions (DFO, 22BP), after normalization to *Tbp* expression levels as the loading control. Mean values with SEM (standard error of the mean) are shown, normalized to the WT control condition. The statistical trends or levels of significance are illustrated by symbols, namely T: 0.1 > *p*>0.05, * or #: *p*< 0.05, ** or ##: *p* < 0.01, *** or ###: *p* < 0.001, **** or ####: *p* < 0.0001. Mutant cells are represented by dashed bars, WT cells by plain colors. Asterisks are used for WT MEF to represent significant effects between treated and untreated control cells, while hashtags refer to *Pink1*^−/−^ MEF with significant differences between treated versus untreated control cells. Genotype-dependent significant differences of *Pink1*^−/−^ versus WT MEF are illustrated by horizontal lines below asterisks. Detailed fold-changes and *p*-values are listed in Supplementary Table S2.

Global proteomics by mass spectrometry usually detects less than 10,000 proteins. However, this represents only a minor fraction of all proteins in MEF cells. The human genome project identified about 20,000 genes, each of which encodes up to 10 proteins, leading to current estimates of 80,000–400,000 proteins within the human body and probably also in the mouse. Proteins with low abundance, integration into membranes, complexes with nucleotide chains may not be detected easily. For a systematic evaluation of all core events in iron homeostasis, we used RT-qPCR to target all relevant factors and assess their transcriptional changes after 16 h in three conditions. Firstly, WT MEF cells were employed in a discovery phase (this paragraph, with Figure 3 and Supplementary Figure S5 illustrating expression adaptations; Supplementary Table S2 provides fold-changes with significance values). Secondly, *Pink1*^−/−^ MEFs were used (subsequent paragraph 2.3) to test reproducibility versus genotype-dependent alterations.

In the discovery phase of this project, we wanted to account for biological variability and screened a large number of WT MEF for iron-dependent expression regulations (plain bars in Figure 3 and Supplementary Figure S5). To test reproducibility, the iron chelator drug DFO was used as an alternative to the iron chelator 22BP. The novel observations regarding transcriptional induction of mitophagy factors *Pink1* and *Prkn* (encoding the protein PARKIN) upon iron depletion is documented in Figure 3A. As further proof-of-principle for the iron-responsiveness of fibroblast expression profiles, Figure 3B shows the transcriptional response of the transferrin receptor (*Tfrc*), which is the main receptor to import iron into the cell. Also shown is the iron storage ferritin chain with its heavy subunit (*Fth1*) and light subunit (*Ftl1*), as well as the small subunit of ribonucleotide reductase (*Rrm2*), as Fe^3+^ binding ferritin superfamily member. *Tfrc* appeared reduced only to 0.45-fold under iron excess conditions (*p* = 0.7155), again suggesting that iron levels in the basal culture medium were already so high that transferrin-receptor expression could not be downregulated much during FAC administration. *Tfrc* was highly induced upon iron deficiency. Thus, the main regulator of iron uptake responded to iron depletion in a sensitive manner. *Fth1* and *Ftl1* were not changed significantly and displayed high variability among the 9 different WT MEF cell lines, but both ferritin subunit transcripts showed about 1.5-fold higher levels during iron excess. Conversely, *Rrm2* as a deoxynucleotide biosynthesis enzyme exhibited significantly lower levels during iron depletion. *Slc40a1* mRNA encoding ferroportin-1 as cellular iron exporter appeared with levels below 0.45-fold during iron depletion (Supplementary Figure S5). These experiments confirmed that MEF cells are responsive to manipulation of iron availability, so we performed further studies into their adaptations of mRNA expression, focusing on all crucial factors of the cellular iron transport and mitochondrial homeostasis, of the heme synthesis pathway and hemeproteins, of the ISC biogenesis pathway and ISC-containing factors.

There were various factors related to these pathways that exhibited no significant dysregulations under any condition or were less relevant or redundant, so they were summarized in Supplementary Figure S5. In alphabetical order, these factors include *Abcb6* (ATP binding cassette subfamily B member 6)*, Aco1* (Aconitase 1), *Aco2* (Aconitase 2), *Alas1* (5′-aminolevulinic acid), *Bach1* (BTB domain and CNC homolog 1), *Bdh2* (3-hydroxybutyrate dehydrogenase-2), *Brip1* (Brca1 interacting protein C-terminal helicase 1), *Cdc42bpa* (Cdc42 binding protein kinase alpha), *Cisd1*, *Cisd2*, *Cp*(Ceruloplasmin), *Cygb*, *Dpyd* (Dihydropyrimidine dehydrogenase), *Elp3* (Elongator acetyltransferase complex subunit 3), *Ercc2* (ERCC excision repair 2), *Fdx1* (Ferredoxin 1), *Fech*, *Flvcr1* (Feline leukemia virus subgroup C cellular receptor 1a), *Fxn* (Frataxin), *Hebp1*, *Myl6*, *Ncoa4* (Nuclear receptor coactivator 4), *Pcbp1*, *Pcbp2*, *Prim2* (DNA primase subunit 2), *Rsad1*, *Rsad2* (Radical S-adenosyl methionine domain containing 1/2), *Rtel1* (Regulator of telomere elongation helicase 1), *Slc25a37, Slc40a1, Steap3,* and *Trf* (Transferrin). The other factors with important dysregulation are shown in the main figures and are individually mentioned in Table S2 as well as the text below, together with their respective roles.

*Tfrc* mRNA is stabilized by IREB2, whose mRNA was also induced after iron deprivation (Figure 3C). After transferrin binding to TFRC protein and subsequent endocytosis of this complex, the acidic pH in endosomes releases ferric iron (Fe^3+^), which is reduced to the ferrous form (Fe^2+^) by the metalloreductases STEAP2 and STEAP3. Iron molecules then get exported by the Divalent metal transporter 1 (DMT1 encoded by *Slc11a2*) to the cytosol [4,72]. After iron overload, *Steap2* and *Slc11a2* mRNA showed a significant average 1.25-fold upregulation (Figure 3C, similar to *Steap3* in Supplementary Figure S5), indicating higher biosynthesis of the factors responsible for the reduction and export of iron. During both forms of iron depletion, *Steap2* and *Slc11a2* mRNAs were significantly reduced to 0.7-fold, so their levels are in direct correlation with iron availability.

The ISC-containing 3-hydroxybutyrate dehydrogenase-2 (BDH2) catalyzes the rate-limiting step in the biosynthesis of siderophores, which are soluble Fe^3+^ binding agents [73]. Upon iron depletion, *Bdh2* transcription also was downregulated to 0.7-fold (Supplementary Figure S5). BDH2 inhibition was shown to result in cellular iron accumulation [74], so again its expression adaptation could represent a homeostatic effort to increase intracellular iron levels.

The poly(RC)-binding-protein-2 (PCBP2) is involved in mRNA metabolism and translation, as well as innate immune signaling, but was previously shown to function also as a chaperone for the LIP in the cytosol and to interact with HMOX1 [75]. However, neither *Pcbp2* nor *Pcbp1* transcripts were consistently changed by iron level manipulation (Supplementary Figure S5). Interestingly, the mRNA of *Pcbp3* was reduced to 0.5-fold after iron depletion, emphasizing its role in iron metabolism as the PCBP family member with the strongest ferritin interaction [76].

The iron regulatory proteins (IRP1 encoded by the *Aco1* mRNA, and IRP2 by *Ireb2*) sense cytosolic iron availability and ensure adequate iron supply to mitochondria [77], firstly via association with iron response elements (IREs) in the untranslated region (UTR) of ferritin *Fth1*/*Ftl1* mRNA to inhibit its translation, and secondly via association with iron importers *Tfrc*/*Slc11a2* mRNA to stabilize them and facilitate their translation when cellular iron levels are low. During both forms of iron depletion, the transcription of *Ireb2* showed a 1.4-fold upregulation, while *Aco1* showed a consistent reduction to 0.6-fold (Supplementary Figure S5). Under conditions of sufficient iron, IRP1 exerts its cytosolic aconitase functions while IRP2 gets degraded, resulting in converse effects with increased ferritin translation and TFRC degradation [78]. After iron overload, we detected no relevant expression adaptation of both transcripts encoding iron regulatory proteins.

Expression of the mitochondrial inner membrane transporter *Slc25a28*, which encodes Mitoferrin-2 (MFRN2), did not react after iron overload but was significantly reduced to 0.6-fold after iron depletion. Mitoferrin-1 (*Slc25a37* or *Mfrn1*), however, did not show altered expression upon iron level manipulation in WT cells (Supplementary Figure S5). MFRN1 forms a complex with the mitochondrial inner membrane iron transporter ABCB10 [72,79]. *Abcb10* transcript levels were 1.4-fold higher under both iron depletion conditions, reaching significance for DFO. Given that it is not clear how these two inner membrane transporters of iron are acting in a complementary fashion, it is interesting to note that *Abcb10* mRNA was higher during low iron conditions, as expected for an iron uptake factor. However, *Slc25a28* mRNA was diminished under the same treatment, a response that would be in line with an iron export factor. In comparison, *Abcb7* and *Abcb8* (ATP binding cassette subfamily B member 7/8) are thought to have mitochondrial export functions [72,80,81,82] and are also important for heme biosynthesis [83]. As expected after iron depletion, *Abcb7* transcript levels were halved, similar to *Abcb8* (Figure 3).

Once transported into the mitochondrial matrix, iron may be stored or used for the biosynthesis of ISC and heme [84]. The mitochondrial iron storage factor mitochondrial ferritin was not detectable in MEF cells under the conditions tested. For biosynthesis purposes, iron is incorporated by FXN into the complex containing sulfur-containing NFS1 (Nitrogen fixation 1 homolog), ISCU (Iron-sulfur cluster assembly enzyme), and LYRM4 (LYR motif containing 4) to generate [2Fe-2S] clusters, which associate with GLRX5 and the assembly factor BOLA1 (BolA Family Member 1) [3,85,86]. Subsequently, these ISC are transferred by the ISC scaffold NFU1 (Nfu1 iron-sulfur cluster scaffold) to target proteins [87]. After both iron depletion conditions, *Glrx5* expression was reduced to 0.5-fold. *Fxn*, *Bola1,* and *Nfu1* levels were around 0.7-fold after iron depletion, but this was not statistically significant. The converse iron overload did not modulate expression of the ISC biogenesis proteins.

Pursuing the heme- and ISC-associated pathways into the cytosol, it is relevant that the putative heme release factor *Pgrmc1* (Progesterone receptor membrane component 1), as a cytosolic factor in association with mitochondrial ferrochelatase, showed a highly significant and more than two-fold transcriptional induction after iron depletion. Heme oxygenase 1 (HMOX1) acts to degrade cytoplasmic heme by cleaving it to biliverdin, as the rate-limiting step of heme breakdown [88]. After iron depletion, also *Hmox1* mRNA was strongly upregulated with high significance. These upregulations might reflect compensatory cellular efforts to recruit iron via heme breakdown. The cholesterol elimination factor CYP46A1 (Cytochrome P450 family 46 subfamily A member 1) belongs to the Cytochrome P450 family, which is known to bind heme as a co-factor [89,90]. After iron depletion, *Cyp46a1* transcript showed a statistical trend towards a 1.5-fold increase; it was not altered after iron overload. Continuing the heme-related pathway, the transcript levels of Cytoglobin (*Cygb*) [91] were quantified, as one vertebrate globin family that is expressed in fibroblasts, but they showed no change and high variability (Supplementary Figure S5).

Not only heme but also ISC are incorporated into target proteins, many of which have nucleotide processing functions. Among the ISC-containing factors present inside and outside of mitochondria, the essential ribosome recycling factor ABCE1 (ATP binding cassette subfamily E member 1) exerts crucial functions to avoid an accumulation of ribosomes at the stop codon, inefficient ribosomal cycling, and stalled translation [92,93]. Interestingly, after both forms of iron depletion, a significant decrease to 0.6-fold was observed for *Abce1* mRNA, while iron overload triggered no expression adaptation.

The ISC-containing phosphoribosyl pyrophosphate amidotransferase (PPAT = GPAT) is the rate-limiting enzyme in de novo purine nucleotide biosynthetic pathways. Its expression showed a strong direct correlation of high significance with the availability of iron. After incubation with FAC, *Ppat* was significantly upregulated 1.5-fold. After iron depletion, it was downregulated with high significance to 0.3-fold levels. Both sensitive adaptations provide evidence that iron is important for nucleotide homeostasis.

The Nth like DNA glycosylase 1 (NTHL1) has relevant functions in base excision repair, harbors an ISC, and is localized both in the nucleus and the mitochondrial matrix. The expression of *Nthl1* reacted similarly to *Ppat* with significant upregulation after iron overload versus downregulation after both forms of iron depletion, although with less effect size and less significance.

Similarly, three other ISC-associated nuclear factors implicated in DNA quality control also showed consistent downregulations after iron deprivation, namely the DNA replication helicase *Dna2*, the DNA primase subunit *Prim2* (Supplementary Figure S5), and the DNA polymerase delta subunit *Pold1*. Also, the ISC-containing Elongator complex protein 3 (ELP3), which acts in tRNA modification [94], showed significantly lower transcript levels to 0.7-fold after iron depletion (Supplementary Figure S5).

The tRNA wybutosine synthesizing protein 5 (TYW5) was reported to catalyze a carbon hydroxylation using Fe^2+^ ions as cofactors, so its activity depends on iron levels. *Tyw5* expression did not react to iron overload, but it was significantly downregulated after iron depletion.

Jointly, these results in WT cells indicate that upon iron overload and even more upon iron depletion, transcriptional expression adapts up to two-fold within 16 h for specific iron homeostasis factors at the plasma membrane, in endosomes and the cytosol, inside mitochondria as well as the nucleus. A negative correlation was found for iron transport components at the plasma membrane and mitochondrial membrane, which responded to iron depletion with induced expression of *Tfrc/Ireb2* and Abcb10, respectively. Similarly, the iron recruitment option via mitochondrial heme release and its cytosolic breakdown responded to iron depletion with induced expression of *Pgrmc1*/*Hmox1*. Furthermore, the increased need for the heme-binding cholesterol catabolism enzyme *Cyp46a1* seemed apparent during iron depletion, since its transcript levels were consistently elevated. In contrast, a strong direct positive correlation with iron levels was observed for endosomal and cytosolic iron processing factors *Steap2* (less for *Slc11a2*), exhibiting increased expression upon iron overload versus decreased expression upon iron depletion. A similarly strong direct correlation was also documented for ISC-containing factors of nucleotide metabolism, namely *Ppat* and *Nthl1*. All other expression adaptations observed simply reflected the diminished synthesis of iron-associated factors under conditions of low iron levels.

### 2.3. Transcriptional Analyses of Expression Adaptations of Pink1^−/−^ MEF to Altered Iron Levels

As a validation effort and to obtain additional mechanistic insights in this project, experiments were performed in 3 *Pink1*-deficient MEF lines where mitophagy is impaired. Again, FAC-mediated iron overload and DFO/22BP-mediated iron depletion were studied. With this approach, we hoped to elucidate how iron, ISC, and heme homeostasis interdepend with mitochondrial turnover.

Figure 3A shows mRNA levels of *Pink1* and *Prkn* as regulators of mitophagy. The bars hatched in black within the first panel confirm the knockout of *Pink1* in the 3 MEF lines and reproduce the high transcriptional induction of *Pink1* after iron deficiency (see also Supplementary Table S2). This transcript induction was much less strong in the *Pink1*^−/−^ cells than in WT cells. These *Pink1*^−/−^ MEF derive from a mouse where an intron is retained in the *Pink1*^−/−^ mRNA and triggers a changed reading frame, so *Pink1* mRNA is rapidly degraded and the PINK1 protein is absent, but the *Pink1* promoter is still actively responding to specific stressors like iron depletion. Similarly, the *Prkn* transcript again got induced upon iron depletion. These results in Figure 3A corroborate the concept that PINK1/PARKIN-dependent mitophagy gets highly induced after iron starvation in MEF. We showed previously in the neuronal cell line SH-SY5Y that nutrient starvation by HBSS medium (which includes transferrin deprivation) will induce *Pink1* and *Prkn* transcript expression and protein abundance together with activation of mitophagy and lysosomal degradation, by the use of recombinant tagged PARKIN-constructs [19].

Overall, the comparison of PINK1-deficient cells, treated versus untreated, reproduced many observations (see significance symbols above hatched bars in Figure 3, numeric values in Supplementary Table S2 and the overall pattern in Supplementary Figure S5): Again, iron depletion induced the mitophagy factors *Pink1*, *Prkn*, the iron import factor *Tfrc*, and factors for iron-release from heme such as *Pgrmc1*, *Hmox1* with *Cyp46a1*, while it reduced the siderophore biosynthesis factor *Bdh2*, the mitochondrial iron homeostasis factors *Slc25a28*, *Abcb7*, *Abcb8,* and *Glrx5*, as well as the nucleotide surveillance factors *Ppat*, *Nthl1*, *Dna2*, *Pold1, Tyw5,* and *Elp3*. Reproducibly, iron excess induced the iron reductase *Steap2*, as well as the nucleotide surveillance factors *Ppat* and *Nthl1*. These findings constitute a validation of the regulations in WT cells.

**Figure 4:**
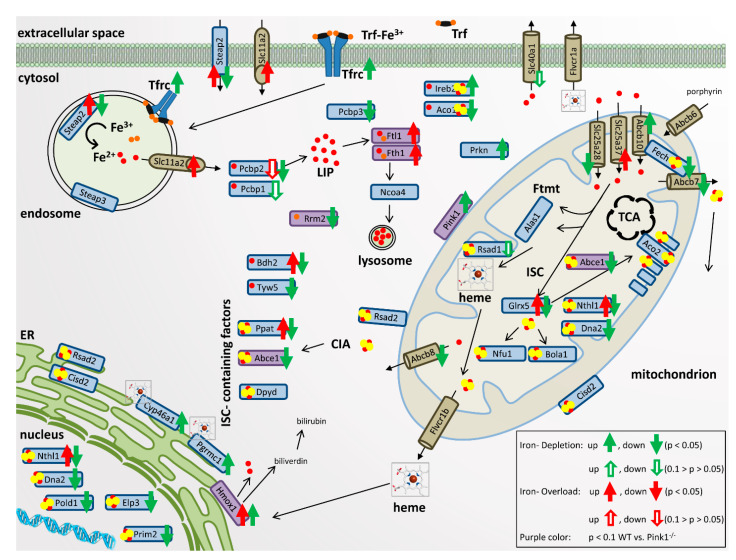
Overview of cellular iron uptake, storage, and export, iron transport and processing, mitochondrial iron homeostasis, heme production and turnover, iron-sulfur-cluster (ISC)-biogenesis and ISC-binding within the cells. For each factor with significant mRNA expression adaptation in room temperature (RT)-qPCR, green arrows indicate the direction of transcript change during iron depletion, while red arrows refer to changes during iron overload. Ferric iron (Fe3^+^) is illustrated with orange dots and ferrous iron (Fe2^+^) with red dots; ISC are represented by red and yellow dot clusters. CIA: cytosolic iron-sulfur-cluster assembly machinery; ER: endoplasmic reticulum; FTMT: mitochondrial ferritin; LIP: labile iron pool; TCA: tricarboxylic acid cycle. Factors with significant genotype-dependent changes with specificity for *Pink1*^−/−^ cells are represented by purple coloring.

It is crucial to note that several regulations lost or reached significance in *Pink1*^−/−^ MEF, compared to WT MEF. A schematic overview of all significant iron effects and trends to regulate the expression of its homeostasis factors at their mRNA levels is provided in Figure 4. Consistently, the cells with PINK1-absence (a state that occurs physiologically when mitochondria are healthy) were less responsive to iron depletion for mRNA adaptations. This rule concerned the plasma membrane *Tfrc*, cytosolic *Ireb2*, *Steap2*, *Bdh2, Abce1,* and mitochondrial *Abcb10* expression; conversely, the cells with PINK1-absence were reacting more strongly to iron excess. This pattern was visible for cytosolic factors *Fth1*, *Ftl1*, *Hmox1* with significance or trend, and similarly without significance for cytosolic *Slc11a2*, *Bdh2, Aco1,* as well as mitochondrial *Slc25a37* and *Glrx5* regulations. These mRNAs may be elevated due to enhanced transcript synthesis or due to enhanced transcript stability. *Fth1*, *Ftl1,* and *Slc11a2* mRNAs have regulatory elements in their 5′ UTR that modulate their translational repression by IRP1/ACO1 and IRP2/IREB2 under influence of the LIP (which is modulated by BDH2), whereas the *Hmox1* mRNA contains regulatory elements to modulate its translational repression under the influence of heme concentrations [95,96]. Furthermore, there are known functional interactions between cytosolic IRP1/ACO1 and mitochondrial SLC25A37 as well as GLRX5 [57,97]. In contrast, most other mitochondrial effects did not depend on PINK1 presence, nor did nuclear effects, except for the significant reduction of *Prim2* upon iron deprivation (Supplementary Figure S5). These observations provide preliminary evidence that PINK1 biosynthesis, which occurs after mitochondrial damage, sends toxicity-limiting signals mainly to the cytosol during iron overload.

The comparison of *Pink1*^−/−^ with WT cells demonstrated five genotype-dependent effects: Strong excess inductions upon iron overload due to *Pink1*-ablation were observed for *Fth1* (1.97-fold, *p* = 0.0291), *Ftl1* (1.64-fold, *p* = 0.0380), and *Hmox1* (2.07-fold, *p* = 0.0562) mRNA, whereas deficient mRNA levels were observed for the ferritin superfamily member *Rrm2* (0.48-fold, *p* = 0.0864) upon iron shortage. Moreover, in untreated cells, a reduction of basal *Abce1* mRNA levels (0.70-fold, *p* = 0.0238) existed in *Pink1*^−/−^ cells a priori. Thus, the main impact of PINK1 on iron homeostasis factors occurred via the ferritin superfamily, which binds ferric ion (Fe3^+^). It is interesting to note that also the global proteome survey documented PINK1 impact on 8 iron homeostasis factors, among which FAC-downregulated MYL6, but also TCIRG1, and PCBP3 protein were implicated in the recruitment of soluble iron to Fe3^+^-binding ferritin. It was previously observed that induction of mitophagy with stabilization of PINK1 protein occurred in parallel to iron liberation via ferritin reduction [98], so the stability of PINK1 versus ferritin appeared conversely regulated, and our finding of PINK1 deficiency to trigger ferritin mRNA upregulation is credible. A minor PINK1 impact targeted heme homeostasis via *Hmox1* mRNA levels, as well as FECH, JMJD6, CYB5A, and GYPC protein abundance. Under untreated conditions, only the PINK1-dependent reduction of GLRX5 concerned the ISC biogenesis pathway, and this may relate to the reduction of basal mRNA levels for the ISC-containing *Abce1.*

### 2.4. Quantitative Immunoblots for Validation and Mechanistic Analyses

**Figure 5:**
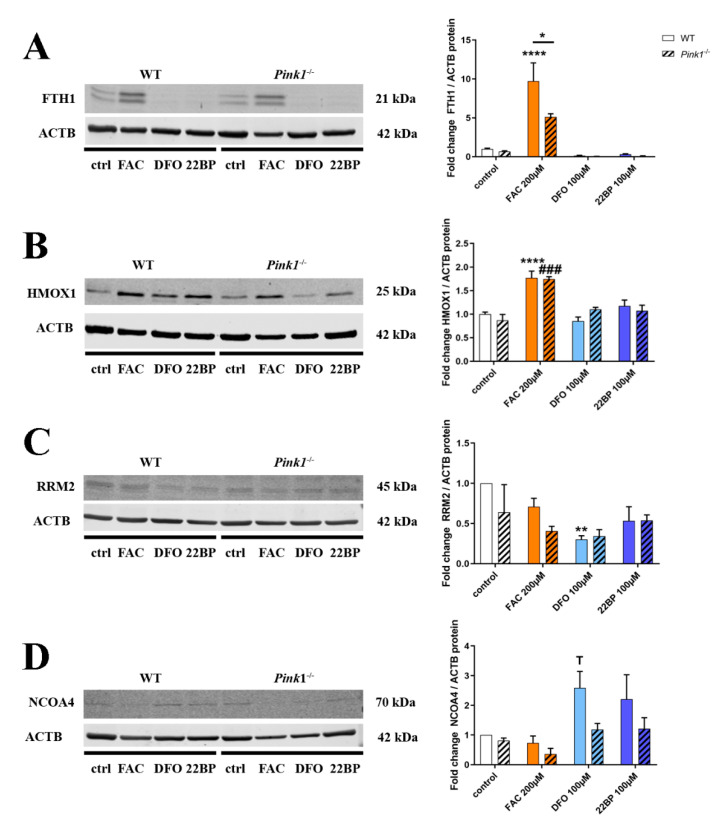
Quantitative immunoblots for (**A**) FTH1, (**B**) HMOX1, (**C**) Ribonucleotide reductase regulatory subunit M2 (RRM2), and (**D**) nuclear receptor coactivator 4 (NCOA4) in WT and *Pink1*^−/−^ MEF, under untreated control conditions (Ctrl), after iron overload (FAC) and after two different iron depletion drugs (DFO, 22BP), administered over 48 h. Protein abundance signals were normalized to beta-Actin levels (Actin beta (ACTB)) as a loading control. The panels on the right show their densitometric quantifications, normalized to WT untreated conditions. WT *n* = 4–6, Pink1^−/−^ *n* = 3, one exemplary set is shown. The statistical trends or levels of significance are illustrated by symbols, namely T: 0.1 > *p*>0.05, *: *p* < 0.05, **: *p* < 0.01, ###: *p* < 0.001, ****: *p* < 0.0001. Mutant cells are represented by dashed bars, WT cells by plain colors. Asterisks represent significance in WT MEF, treated versus untreated control, while hashtags refer to *Pink1*^−/−^ MEF, treated versus untreated control. Genotype-dependent significant differences of *Pink1*^−/−^ versus WT MEF are illustrated by horizontal lines below asterisks.

Now we wanted to assess in further validation experiments if the observed changes are also significant at the protein level upon analysis of additional MEF lines (Figure 5), focusing on three dysregulated factors whose transcript dysregulation had marked effect size (about two-fold up or down) and where a promising commercial antibody was available.

In the case of FTH1, a 9.72-fold (*p* < 0.0001) strongly significant induction after FAC was found in RIPA-extracted proteins from WT cells (Figure 5A) (in further SDS-extractions of sample pellets no specific immunoblot band was detected), but in contrast to the findings at mRNA level this FTH1 protein induction was significantly diminished in *Pink1*^−/−^ cells (*p* = 0.0476). Although this downregulation had not been significant in the mass spec analysis or the immunoblot validation for 3 mutant versus 3 WT cells, the effect became clear when more WT cell lines were investigated. This finding is in good agreement with previous literature that could not document increased ferritin abundance in PD brains despite the iron accumulation [99,100,101]. As for *Pink1*^−/−^ MEFs here, immortalized endometriotic cells were previously reported to respond to ever more excessive iron dosage with progressively lower ferritin protein levels and a converse increase of LC3A within ATG5/ATG7-dependent autophagy [102]. If ferritin-producing glia cells in a PARK6 brain cannot respond adequately to iron excess, then this weakness of antioxidant defenses might debilitate neurons and glia, leading to compensatory storage of iron overload in neuromelanin granules, and exhausting the storage capacity of neurons.

In the case of HMOX1 (Figure 5B), a strongly significant induction after FAC was found in WT cells (1.77-fold, *p* < 0.0001), and the analysis of *Pink1*^−/−^ cells did not confirm a genotype-dependent increase at the protein level. Thus, the hypersensitive mRNA induction after FAC in *Pink1*^−/−^ MEF might represent a compensatory effort to ensure sufficient protein abundance in a period of high turnover. Previous work reported the HMOX1 turnover to depend on ubiquitin/proteasome pathway inhibition by MG-132, and a PINK1 loss-of-function mutation decreased HMOX1 abundance in these experiments [103], so HMOX1 protein stability in *Pink1*^−/−^ cells after FAC might also be reduced upon analysis of more cell lines.

Regarding RRM2 (Figure 5C), a significant downregulation after DFO in WT cells was documented, in good agreement with proteome and transcript data.

To test mechanistically whether PINK1 as a master regulator of mitochondrial autophagy could also influence ferritin degradation via autophagy, quantitative immunoblots assessed the levels of the ferritinophagy adaptor protein NCOA4 (Figure 5D). As expected for the liberation of iron from reserve pools during iron-shortage, a 2.59-fold NCOA4 induction after DFO treatment was observed in WT cells, reaching a statistical trend despite strong clonal variability (*p* = 0.0593). In *Pink1*^−/−^ cells this NCOA4 upregulation for ferritinophagy appeared diminished (to only 1.19-fold, *p* = 0.9432), but certainly not augmented, and these effects did not reach significance upon analysis of 3 KO versus 4 WT lines (*p* = 0.1445). Overall, these data in Figure 5D do not support the notion of an excessive ferritinophagy after FAC as an explanation for the abnormally low FTH1 protein upregulation in *Pink1*^−/−^ cells (Figure 5A). We considered whether PINK1 phosphorylation might influence FTH1/FTL1/HMOX1 protein stability independent of autophagy/proteasome degradation, similar to its activating/stabilizing influence on Ubiquitin and Parkin [104]. To first ensure a deeper understanding of the mechanisms governing iron regulation and their modification by PINK1, we analyzed the data further to understand the adaptations of relevant nuclear transcription factors, and the responses of post-transcriptional control by IRP1/IRP2 over ferritin mRNA translation repression.

### 2.5. Regulation of Relevant Transcription Factors with Their Targets Is Subtly Modified by PINK1

**Figure 6:**
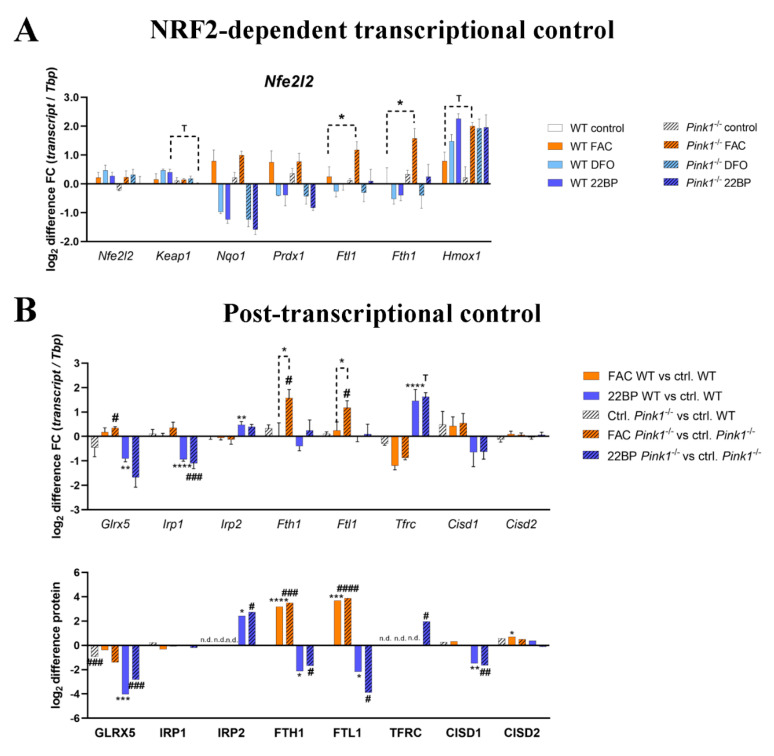
Adaptation to changing iron concentrations for key factors in (**A**) the Nuclear factor, erythroid 2 like 2, Nfe2l2 (NRF2) transcription factor complex with its modulator Kelch-like ECH associated protein 1 (KEAP1) and their downstream transcript targets, as well as (**B**) the post-transcriptional control of iron import and storage. For mRNA expression analysis, the *Tbp* levels were used as loading control and after normalizing the fold changes to control conditions in WT MEF, the log_2_ fold changes of individual fold changes were calculated. The fold change of protein abundance label free quantification (LFQ) value was also represented as log_2_ difference. Mutant cells are represented by dashed bars, WT cells by plain colors. Asterisks represent significance in WT MEF, treated versus untreated control, while hashtags refer to *Pink1*^−/−^ MEF, treated versus untreated control. To simplify the overview, in (**A**) only genotype-dependent significances are shown, whereas (**B**) includes all significances with respect to control conditions. Detailed *p*-values and individual fold changes can be seen in Supplementary Tables S1,2. The statistical trends or levels of significance are illustrated by symbols T: 0.1 > *p*>0.05, * or #: *p* < 0.05, ** or ##: *p* < 0.01, *** or ###: *p* < 0.001, **** or ####: *p* < 0.0001.

To define the relevant nuclear events governing the transcriptional responses, those transcription factors that had shown abnormal abundance in the proteome profile, and other relevant transcription factors—both, for the antioxidant response to FAC and the hypoxic response to iron chelators—were assessed also in their rapid mRNA regulation by the highly quantitative RT-qPCR method (Figure 6A, Supplementary Figure S6), together with their best-established transcription targets.

As a coordinator of hypoxic transcription efforts, HIF1A activity was previously found to be stabilized during PINK1 deficiency [105], suggesting a negative correlation between both factors. As coordinator of antioxidant transcription efforts, NRF2 (encoded by the *Nfe2l2* gene) activity was previously reported to enhance *Pink1* expression, and to accumulate and translocate to the nucleus when PINK1 induced and stabilized [106,107], suggesting a positive correlation between these two factors. NRF2 is known to enhance the transcription of *Fth1*/*Ftl1*/*Hmox1* during ferroptosis [108,109], so a hypothetical NRF2 compensatory hyperactivity upon PINK1 absence might explain excessive transcription inductions after FAC. The NRF2 inhibitor KEAP1 is degraded by autophagy [110], therefore altered autophagy due to PINK1 absence might feedback onto NRF2 activity.

Indeed, for transcription factor TFEB, as a coordinator of autophago-lysosomal pathway activity (Supplementary Figure S6A), in untreated *Pink1*^−/−^ cells a significant elevation of mRNA levels suggested augmented activity of general autophagy, perhaps in compensation of the selective mitophagy deficit. During iron depletion, WT cells efficiently induced *Tfeb* mRNA, while *Pink1*^−/−^ cells were unable (Supplementary Figure S6A). However, these changes appeared to only have subtle impact, given that downstream TFEB targets *Ctsd*/*Ctsf*/*Sqstm1* did not exhibit significant genotype-dependent expression effects. Transcription factor MITF as an additional coordinator of autophago-lysosomal activity (Supplementary Figure S6B) showed a consistently stronger expression induction after FAC, but *Mitf* mRNA and its downstream target *Ctsb* exhibited no genotype-dependent expression adaptations.

NRF2 encoding *Nfe2l2* mRNA levels showed no regulation at all (Figure 6A), but its inhibitor *Keap1* showed a trend towards reduction after 22BP, in good agreement with its protein levels in Figure 2B and Supplementary Figure S2B. Among the downstream targets of NRF2/KEAP1, several factors like *Gpx1*/*Gpx3* showed no upregulation after FAC, other factors like *Nqo1*/*Prdx1* showed induction after FAC and reduction after DFO/22BP without any genotype-dependent modulation, and only *Fth1*/*Ftl1*/*Hmox1* showed the FAC-triggered hypersensitive induction in *Pink1*^−/−^ cells, so NRF2 alone does not explain the response pattern.

JUND (Jun proto-oncogene) is a component of antioxidant NRF2 transcription factor complexes [111], but did not exhibit induced expression after FAC, instead, it showed strong consistent mRNA induction after iron chelation (Supplementary Figure S6C), consistent with the upregulation of JUND protein after 22BP in Supplementary Figure S2B. Its downstream target *Mmp14* in *Pink1*^−/−^ cells showed a trend to stronger reduction after 22BP, in good agreement with the downregulated MMP14 protein abundance after 22BP in Supplementary Figure S2B, as a converse regulation to MMP14 protein upregulation after FAC in Figure 2B.

*Hif1a* mRNA as coordinator of hypoxic transcription responses showed no relevant expression regulation (Supplementary Figure S6D), despite the upregulation of HIF1A protein after 22BP in Figure 2B and Supplementary Figure S2B. Its upstream regulator EGLN1 (Egl-9 family hypoxia-inducible factor 1) [112] showed strong and consistent expression inductions after iron chelation, in agreement with EGLN1 protein upregulation in Figure 2B and Supplementary Figure S2B. For their downstream targets *Hk1* (Hexokinase 1)/*P4ha2* (Prolyl 4-hydroxylase subunit alpha 2)/*Nos2* (Nitric oxide synthase 2), iron shortage triggered clear mRNA inductions as the basis of their increased protein abundance in Figure 2B and Supplementary Figure S2B. Overall, despite the very clear activation of this hypoxia pathway after iron deprivation, there were no PINK1-dependent effects in this pathway whatsoever. The upregulation of FOXO3 protein after 22BP in Figure 2B was also clearly reproduced at the mRNA level (Supplementary Figure S6E), but interestingly this expression induction was significantly diminished in *Pink1*^−/−^ cells. Again the impact on downstream targets like *Bnip3* (BCL2 interacting protein 3)/*Gabarapl1* (GABA type A receptor-associated protein-like 1) was too subtle to trigger genotype-dependent effects there.

Regarding the hypoxic transcription factor MEF2D (Myocyte enhancer factor 2D) and its upstream splicing modulator RBFOX2, the expression modulations were small, but in the case of their downstream targets *Homer1*/*Jmjd6*/*Rrm2,* the expression modulations by iron deprivations were strong and consistent. PINK1-deficiency was found in Supplementary Figure S2E to trigger RBFOX2 protein upregulation, and indeed genotype-dependent effects were observed among the mRNA targets *Jmjd6* (another splice modulator) and *Rrm2*, in both cases with a trend towards reduced transcript levels in *Pink1*^−/−^ cells (Supplementary Figure S6F).

Overall, iron modulates the expression regulation of transcription factors and their mRNA targets in good agreement with global proteome profiles, particularly for the hypoxia pathway after iron chelator treatment. In contrast, the PINK1-dependent effects on transcriptional control were only subtle, with a consistent change in several downstream targets being documented only for the hyper-reactivity of *Fth1*/*Ftl1*/*Hmox1* in the antioxidant NRF2/KEAP1 pathway, while other NRF2 targets did not show this regulation pattern.

### 2.6. Regulation of the Post-Transcriptional Control over Iron Homeostasis Is Modified by PINK1

Global proteome and RT-qPCR data were then re-examined in order to assess, to what degree post-transcriptional control mechanisms play a role and are modulated by PINK1 (Figure 6B). The activities of iron-regulatory proteins IRP1/ACO1 and IRP2/IREB2 depend on the stability of their [4Fe-4S] clusters, which are generated in mitochondria from [2Fe-2S] clusters that bind to GLRX5, to then be processed further [113]. When the ISC are not available for IRP1/IRP2 or are damaged, the mRNA for *Tfrc* will be stabilized to increase iron import, while repression of mRNA translation will occur for ferritin heavy and light subunits to maximize iron liberation [95]. Some repair of IRP1 can occur via the [2Fe-2S] clusters of CISD1/CISD2, which protect the cells from ferroptosis [114,115].

Iron overload triggered a stronger *Glrx5* mRNA induction in *Pink1*^−/−^ cells, possibly as a compensatory effort for the significant decrease of GLRX5 protein already at untreated condition as well as after FAC in *Pink1*^−/−^ cells, while iron shortage reduced GLRX5 at mRNA and protein level in all cells (Figure 6A,B).

Also for *Aco1/Irp1*, iron overload triggered a stronger RNA induction in *Pink1*^−/−^ cells, while iron shortage again reduced *Aco1* at mRNA level in all cells, in both conditions these transcript regulations maintained normal protein levels.

FAC treatment had no impact on IRP2 mRNA *Ireb2*, which is rapidly degraded under normal iron concentrations so that no IRP2 protein was detectable. 22BP treatment resulted in marked stabilization of *Ireb2* mRNA, and more than 2-fold upregulation of the protein, as previously shown.

For the ferritin heavy and light chain, after FAC the mRNA induction was hyper-reactive in *Pink1*^−/−^ cells as described previously, while the protein levels appeared similarly upregulated during iron overload, and similarly downregulated after iron deprivation, in this analysis of only 3 WT versus 3 mutant MEF lines by mass spectrometry.

The transferrin receptor exhibited mRNA downregulation under iron excess and upregulation under iron shortage, its protein abundance was detectable and high only in *Pink1*^−/−^ cells during iron shortage, as a possible correlate of an increased iron import need of cells affected by Parkinsonian pathogenesis.

Regarding the IRP1-repair factors CISD1/CISD2, after FAC an upregulation for CISD2 protein was detected, whereas CISD1 protein was downregulated after 22BP. A clear genotype-dependent effect was not observed. The mRNAs of *Cisd1* and *Cisd2* did not show significant changes, although *Cisd1* levels appeared lower during iron shortage.

### 2.7. PINK1-Dependent Effects on Expression in Human Skin Fibroblasts

**Figure 7:**
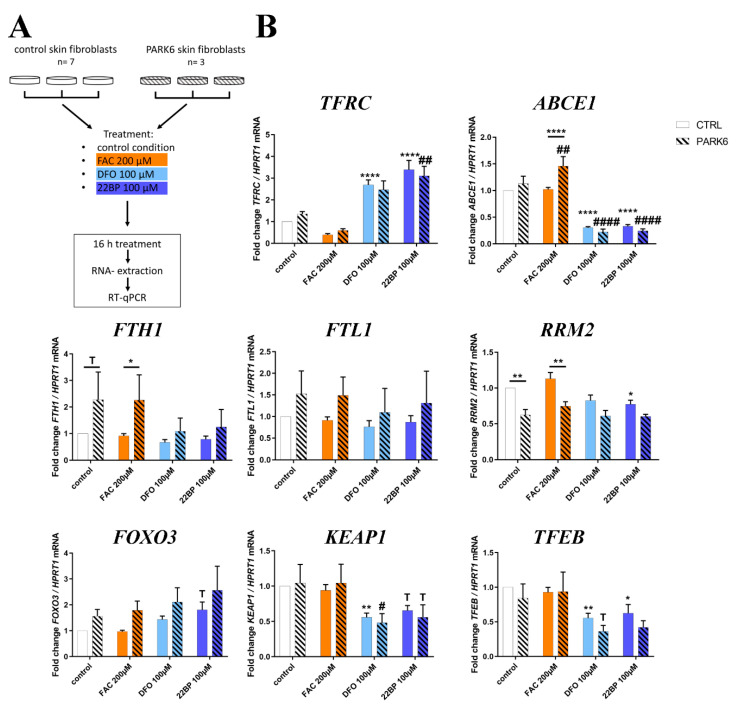
(**A**) Schematic representation of the experimental setup. Human fibroblasts of healthy controls (*n* = 7) and PARK6 patients (*n* = 3) were either left untreated or incubated with FAC, DFO, and 22BP for 16 h, and the extracted RNA was analyzed by RT-qPCR. (**B**) Changes of mRNA expression for key iron homeostasis factors, and crucial downstream effects, which had been significantly dysregulated in previous mouse experiments. Expression levels were normalized against *HPRT1* mRNA as a loading control. Mean values with SEM are shown, normalized to the untreated control condition. The statistical trends or levels of significance are illustrated by symbols T: 0.1 > *p*>0.05, * or #: *p* < 0.05, ** or ##: *p* < 0.01, **** or ####: *p* < 0.0001. Asterisks represent significant changes in control fibroblasts, treated versus untreated control, while hashtags refer to *PARK6* patient cells, treated versus untreated. Genotype-dependent significant differences between patients and controls are illustrated by horizontal lines below asterisks.

To test whether the main effects observed in MEF can be reproduced also in human adult skin fibroblasts, we used cells from 7 healthy individuals versus 3 PARK6 patients with G309D-PINK1 mutation (experiment design in Figure 7A) that were previously shown to exhibit (i) lipid peroxidation, (ii) apoptotic vulnerability, (iii) expression dysregulation of the ferrireductase alpha-synuclein and the kinase LRRK2 (responsible for the PARK1/4 and PARK8 variants of PD, respectively), and (iv) abnormal mitoribosomal translation due to ABCE1 function impairment [14,17,116,117,118,119,120,121,122,123].

In good agreement with the mouse data, the analysis of iron dosage impact on mRNA expression levels by RT-qPCR demonstrated *ABCE1* transcripts significantly and consistently reduced after DFO/22BP in WT and mutant cells (see Figure 3C), whereas they were induced after FAC selectively in mutant cells (Figure 7B). This genotype-dependent upregulation again may be because ABCE1 protein contains an iron-sulfur-cluster and its instability upon oxidative/nitrosative stress has to be compensated by antioxidant compensatory efforts in the patient cells.

The converse regulation was observed for *TFRC* mRNA, where the expected negative correlation with iron led to a consistent increase after DFO/22BP versus a decrease after FAC.

In the case of *FTH1* mRNA, the PINK1-dysfunction triggered a significant excess induction (Figure 7B), validating our analogous observation in MEFs. It is noteworthy that the *FTH1* mRNA showed a trend to increased levels already in the control medium (which has maximized non-toxic iron levels) in cells from adult PARK6 patients, a feature that was not detected in embryonic fibroblasts from mice. For *FTL1* mRNA, generally increased levels were observed in PARK6 cells, but without significance due to high variation among the mutant cell lines. The ferritin superfamily member *RRM2* mRNA exhibited a significant decrease after 22BP, as in mouse cells. It also showed significant genotype-dependent regulations in control medium and after FAC, both levels being decreased for mutant cells, in contrast to *FTH1*. Thus, also in the human organism PINK1 preferentially impacts the NRF2-dependent antioxidative response to iron overload.

There were no genotype-dependent effects for the other factors studied, but iron deprivation tended to upregulate *FOXO3* mRNA levels as observed in mouse, while significantly and consistently downregulating *KEAP1* and *TFEB* mRNA (Figure 7B), two observations that are opposite to the upregulations observed in embryonic mouse cells (Figure 6A). Age-dependent changes in the nuclear transcription regulation of autophagy may help to explain this contrast for both factors.

## 3. Discussion

Our data represent a systematic pioneer effort to document physiological regulations of the global proteome and transcript levels for most factors with relevance to iron, ISC, and heme homeostasis, upon acute changes in iron availability. The fibroblast data were also used to explore molecular mechanisms, how PINK1 mutations trigger the iron brain accumulation that is seen in PD. The global proteome profiles confirmed the well-known converse regulation of both ferritin chain subunits after 22BP versus FAC, and newly identified a converse regulation for the iron-binding purine degradation enzyme XDH, the mitochondrial lipid transporter CPT1A, the collagen degrading factor MMP14, and the glycogen mobilizing enzyme PYGL (only the latter showed an indirect correlation with iron availability). In contrast, the heme oxygenase HMOX1, the nuclear transcription modulator CREG1, and the ferritin superfamily member and nucleotide synthesis enzyme RRM2 showed optimized abundance at untreated conditions, while 22BP more than FAC treatment both triggered upregulation of HMOX1 and CREG1 protein, versus downregulation of RRM2 protein. The targeted mRNA quantification studies of mouse and human fibroblasts showed very good reproducibility, regarding the induction of both ferritin subunits by FAC at the transcriptional level, which exacerbates upon the presence of PINK1 mutations. With high consistency between mouse and human fibroblasts, iron deprivation decreased the expression of *ABCE1* and *RRM2* mRNA, as components of nucleotide synthesis/surveillance pathways.

Iron chelators trigger a transcriptional induction of the mitophagy-mediators *Pink1* and *Prkn* in MEF, according to our novel observations. This is an important finding to explain the previous reports in *C. elegans* that mitophagy is induced upon iron shortage [11,12]. A similar induction of *Pink1* and *Prkn,* accompanied by increased autophago-lysosomal degradation of mitochondria, was also reported in human neuroblastoma cells upon deprivation from fetal calf serum, which contains transferrin as the key supplier of iron during cell culture [19]. Interestingly, iron shortage also leads to a consistent upregulation of beta-synuclein protein (SNCB), an antagonist of alpha-synuclein. While SNCB was shown to have some ferrioxidase activity, alpha-synuclein is known as a ferrireductase [124,125,126,127], similar to STEAP3 [128], and has a physiological localization at vesicles/endosomes and at the interface between mitochondria and endoplasmic reticulum [129]. In the context of human Parkinson pathogenesis, it is noteworthy that the main driver of neurodegeneration in PD, namely the excessive dosage and aggregation of the protein alpha-synuclein, can be modulated by iron via direct binding to an IRE in its mRNA 5′UTR as well as direct binding to the encoded protein [130,131]. Although the upregulation of SNCB after iron deprivation raises the question if its antagonist SNCA is upregulated after iron excess and perhaps influenced by PINK1 similar as *Fth1/Ftl1* mRNA at their 5′UTR hairpins via IRP1/IRP2, it is not possible to investigate the connections between iron and alpha-synuclein further in MEF due to its low abundance in these cells. Experiments in primary neuron cultures will be necessary. However, it is noteworthy that a global transcriptome survey via oligonucleotide microarrays detected an 8-fold induction of alpha-synuclein transcripts in human fibroblasts from PD patients with *Pink1* mutation [119]. Certainly, the observation of increased lipid peroxidation in these PARK6 patient fibroblasts suggests that an enhanced vulnerability for ferroptosis may be present in these cells [17]. Thus, both the negative correlation of iron with *Pink1*/*Prkn* expression and its positive correlation with alpha-synuclein aggregation may contribute to its toxicity, modulating mitophagy, and the neurodegenerative process in PD.

Iron deprivation conversely downregulated RRM2, an effect that will lead to reduced dNTP availability for mtDNA repair, and at the same time to increased superoxide release from uncoupled mitochondria as a cause for mtDNA damage, according to two previous studies [132,133]. This is highly important, given that excessive damage to mitochondrial DNA in the PD brain tissue is a well-established finding [134]. The global proteome profiles, illustrated in Figure 2 and Supplementary Figures S1–S4, and the transcript quantification of iron-binding factors, illustrated in Figure 3 and Figure 4, documented that decreased abundance and expression of nucleotide synthesis/surveillance factors after iron deprivation is a widespread feature, which has to be taken into account when iron chelators will be tested for the neuroprotective therapy of PD.

With consistency between WT and mutant cells, iron shortage upregulated the plasma membrane iron import factor *Tfrc* mRNA together with *Ireb2* as a stabilizer of its mRNA [135]. Iron depletion also upregulated the mitochondrial membrane iron import factor *Abcb10*. In contrast to this avid activation of iron recruitment proteins upon iron deficiency, the expression of intracellular iron disposal factors was in a positive correlation with iron availability. This iron-dependent expression was particularly strong for the endosomal factors STEAP2 and DMT1 (encoded by *Slc11a2*), and exceptionally significant for *Ppat, Nthl1, Dna2, Pold1, Tyw5,* and slightly for *Prim2.* All of these factors contain ISC, and importantly, they act as components of RNA/DNA surveillance. NTHL1 is a DNA N-glycosylase that is mainly localized in the nucleus but also found in mitochondria [136]. It catalyzes the first step in base excision repair and binds a [4Fe-4S] cluster [9]. *Nthl1* mRNA seemed to react very sensitively with a reduction to almost 50% upon iron depletion and induction to 135% after iron overload. PPAT belongs to the purine/pyrimidine phosphoribosyltransferase family and catalyzes the first step of de novo purine nucleotide biosynthetic pathway. It also possesses a [4Fe-4S] cluster, which is needed for protein maturation [9]. PPAT maturation and subsequent function are affected in the neurodegenerative disorder Friedreich Ataxia. This disease is caused by loss-of-function mutations in the *Fxn* gene encoding the frataxin protein, which starts the ISC biogenesis within mitochondria [137,138]. Thus, the transcript reductions of *Nthl1* and *Ppat* probably reflect limited ISC supply from mitochondria that causes impaired maturation/stability of both proteins [139].

All other factors with a downregulated expression upon iron deficiency are either involved in the complex synthesis of ISC or contain one or several ISC. Among them were *Bola1*, *Nfu1,* and *Glrx5*, which play roles in the synthesis of ISC and are localized inside mitochondria. The cytosolic tRNA modification factor TYW5 associates with iron and was also clearly reduced after iron deprivation. A downregulation was noted for ISC-containing cytosolic IRP1/*Aco1* as well as for ISC-containing *Rsad1* (in PINK1-deficient cells), which encodes a heme chaperone in the mitochondrial matrix [140]. However, ISC-containing FECH in mitochondria did not show relevant expression dysregulation. This suggests that ISC homeostasis is not entirely disrupted upon depletion of extracellular iron or in the absence of *Pink1*, but instead, specific vulnerabilities exist, in particular for RNA/DNA quality and genome stability through *Ppat/Nthl1/Dna2/Prim2/Pold1* deficiency. This is a central finding, since DNA integrity is crucial for the healthy lifespan [141], and since the DNA repair pathway was recently identified as the most important modifier of onset age and progression velocity in neurodegenerative diseases [142,143,144]. Jointly, all these data underline the importance of iron for the RNA/DNA quality surveillance, inside and outside mitochondria.

Further mRNA inductions in response to iron deprivation included *Ireb2*/IRP2 as a stabilizer of *Tfrc* mRNA [135], the more than 2-fold induction of the heme-release factor *Pgrmc1* [145], and a massive 4- to 5-fold induction of *Hmox1* (the rate-limiting enzyme in heme-degradation) in response to 22BP, as another possible pathway of iron recruitment. The previously observed impaired *Hmox1* induction after oxidative stress damage in *Pink1*-depleted cells [103] was not detected in MEF in the absence of stress factors. Iron depletion probably increases HMOX1 via HIF1a induction (a well known response to iron depletion) and FAC causes oxidative stress, probably leading to Nrf2 induction and an increase in HMOX1 via this pathway. It is interesting to note that iron-starved cells trigger strong induction of the mitochondrial ferrochelatase-associated heme-release factor *Pgrmc1*, but not a similarly strong effort to export ISC from mitochondria through *Abcb7* induction. The novel observation that iron depletion induces *Cyp46a1* as the rate-limiting enzyme of cholesterol degradation provides a hint of why iron-deficiency anemia patients show lower blood cholesterol levels, and how iron influences steroidogenesis [146,147].

Current knowledge proposes iron deficiency or hypoxia to act via nuclear HIF-1/FIF-2a/HIF-b, triggering a transcriptional induction of *Tfrc* together with *Hmox1*, *Slc11a2* (DMT1), *Slc40a1* (FPN1), *Epo,* and *Cp* [72]. However, in acutely iron deprived MEF, we observed a strong upregulation only for *Tfrc* and *Hmox1*, which contrasted with mild downregulation for *Slc11a2* and *Slc40a1*.

Under untreated conditions, significant dysregulation with *Pink1*^−/−^ genotype-dependence was documented for the ISC-biogenesis factor GLRX5 and for the mRNA of ISC-associated ABCE1 (Supplementary Figure S2E, Figure 3 and Figure 4, also after 22BP in Figure 2B). ABCE1 is one of the most conserved proteins in evolution and is expressed in all organisms except eubacteria. Because of its fundamental role in translation initiation, ribosome biosynthesis and/or ribosome recycling, ABCE1 is essential for life. The reduced ABCE1 function in PARK6 patient fibroblasts was already documented by our previous work to impair the ribosomal translation of mitochondrial precursor proteins, triggering widespread dysfunction for most mitochondrial pathways [122,124]. Our novel mammalian data confirm that ABCE1 has a unique link to the mitophagy factor PINK1, as was observed previously also in *D. melanogaster*. These experiments in flies demonstrated that nucleus-derived mRNAs encoding mitochondrial precursor proteins, such as the complex-I 30kD subunit continuously translated at the outer mitochondrial membrane surface, may be damaged and stall the ribosomal translation machinery during stress periods. This leads to a toxic C-terminal extension of certain amino acids non-coded by mRNA template. In a PINK1-dependent manner, this recruits co-translational quality control factors for RNA/proteins in a process named MISTERMINATE, and triggers mitophagy. During this mitochondrial surveillance process, NOT4 (Ccr4-Not transcription complex subunit 4) generates poly-ubiquitin signals on the co-translational control protein ABCE1, thus attracting autophagy receptors to the mitochondrial outer membrane and contributing to mitophagy initiation [122,124,148]. Thus, fly mitophagy is regulated together with proteostasis in PINK1-dependence via ABCE1. It is relevant to note that longevity is increased upon several genetic perturbations of mRNA translation within the mTOR pathway (Mechanistic target of rapamycin kinase) in yeast, *C. elegans,* and *D. melanogaster* and by mutations that slow down the expenditure of cellular energy by ribosome biogenesis [149,150]. Therefore, it will be interesting to assess the lifespan effects of different ABCE1 mutations in the future.

Strong homozygous depletion of the ABCE1 homolog pixie in *D. melanogaster* results in early lethality [151]. In addition, the homozygous mouse knockout of *Abce1* is embryonically lethal, according to the International Mouse Phenotyping Consortium (IMPC) (https://www.mousephenotype.org/data/genes/MGI:1195458), the international mouse phenotyping consortium. As an ATPase (Adenosintriphosphatase), ABCE1 is responsible for the splitting of the two ribosomal subunits and is thus important for translation termination in mammalian cells, yeast and archaea. To fulfill these roles, it harbors two essential diamagnetic [4Fe-4S]^2+^ clusters [152]. The depletion of *Abce1* was reported to induce the accumulation of ribosome-associated isolated mRNA-3′-UTRs, consistent with a model of ribosome stalling [153]. It is also known as RNaseL-inhibitor and exerts selective control over the stability of mitochondrial mRNAs during interferon-alpha responses to infection [154]. Decreased ABCE1 protein levels were caused by the induction of ROS and this was attributed to the chelation of iron with subsequent loss of stability of ABCE1, indeed its yeast ortholog Rli1 was reported to be crucial for the growth suppression by ROS [93,153]. Interestingly, *Abce1,* as well as *Hbs1l* (Hbs1 like translational GTPase) transcript levels, were shown to be upregulated in the brains of PD patients [148]. Hbs1L is a member of the GTP-binding elongation factor family and was reported to be involved in the regulation of fetal hemoglobin levels [155]. These observations are in accordance with the increased transcript levels of *ABCE1* after iron overload in human *PARK6* fibroblasts (Figure 7).

Selectively after iron overload, which is known to trigger mitochondrial reprogramming and oxidative/nitrosative stress [156], *Pink1*-ablation was observed to induce expression upregulations of several cytosolic iron homeostasis factors. A genotype-dependent trend towards upregulation was observed for *Hmox1* as a factor that protects against heme cytotoxicity. Crucial significant and consistent upregulations appeared after FAC treatment in *Pink1*-ablated cells for *Fth1* and *Ftl1*, the two subunits of the ferritin chain [157,158]. The hyper-responsive induction of *Fth1*/*Ftl1*/*Hmox1* in *Pink1*^−/−^ cells was mirrored by a similar pattern for the ISC-pathway member *Glrx5* and for the iron-regulatory *Aco1* mRNA, perhaps pointing to joint regulation within the same stress response pathway. These FAC-triggered hyper-reactive transcriptional responses of *Fth1*/*Ftl1*/*Hmox1* have NRF2 in common as a transcription factor, but other transcript targets of NRF2 that are not involved in iron homeostasis do not show this regulation pattern, so additional mechanisms must be involved. Despite this transcriptional induction, the ferritin chain heavy subunit protein was significantly decreased in *Pink1*-ablated cells upon immunoblot studies. The repression of *Fth1* and *Ftl1* mRNA translation is a well-established regulation with a physiological role during iron shortage, mainly mediated by the incorporation of mitochondria-generated ISC into the IRP1 and IRP2 proteins. When the cells are iron deprived and ISC cannot be produced, then IRP1 and IRP2 will repress ferritin biosynthesis post-transcriptionally. It is conceivable that this repression occurs erroneously also when mitochondria are dysfunctional and unable to provide ISC. Although the global proteome profile of MEF quantified less than 4000 out of 80,000–400,000 existing cellular proteins, and more exhaustive studies should be done on all proteins of the mitochondrial fraction, our data show several preliminary hints that ISC unavailability/instability due to mitochondrial dysfunction in *Pink1*^−/−^ cells might explain the abnormal regulation of iron homeostasis. Firstly, the cytosolic ISC-repair factor CISD2 increased after FAC (Figure 2A), suggesting an instability of ISC-proteins under these conditions. It is relevant to note that the genetic ablation of CISD1 in mouse causes neurodegeneration with Parkinsonian phenotypes [159], and that iron shortage in MEF cells triggered CISD1 deficiency. Secondly, *Aco1* mRNA encoding IRP1 appeared induced in *Pink1*^−/−^ cells after FAC treatment (Supplementary Figure S5), thus maintaining the steady-state protein abundance normal, but possibly the ratio between the ISC-containing ACO1 holoenzyme versus the ISC-deficient IRP1 apoenzyme shifted. Thirdly, the selective upregulation of TFRC protein in *Pink1*^−/−^ cells after 22BP, together with the hyper-responsive induction of the mitochondrial iron transporter *Slc25a37* (Mitoferrin-1) after FAC in *Pink1*^−/−^ cells (Supplementary Figure S6), might both represent a compensatory effort to maximize iron import because of a mitochondrial impairment of iron utilization, e.g., in the ISC and heme biosynthesis pathways. Fourth, the mitochondrial ISC-biosynthesis factor GLRX5 was decreased to 0.52-fold in absence of treatment in *Pink1*^−/−^ cells, and to 0.77-fold after FAC in WT cells (Supplementary Figure S2E). In human non-hematopoietic cells with GLRX5 deficiency, the reported consequences include a deficit of mitochondrial ISC biogenesis, increased IRP1-mediated repression of ferritin mRNA translation, and elevated TFR1-mediated iron import with subsequent accumulation of cytosolic/mitochondrial iron [57]. It was also observed that cells with GLRX5 deficiency show abnormally high LIP, repressed ferritin levels, and an augmented vulnerability to ferroptosis [160], as appears to be the case also for *Pink1*^−/−^ cells. Notions that the mitochondrial dysfunction inherent in PD leads to progressively conspicuous efforts to maximize ISC-biogenesis are also supported by a report that the abundance of the ISC export factor ABCB7 increased abnormally over time in the neurotoxic MPTP mouse model of PD [161]. The concept that this mitochondrial dysfunction in PD leads to abnormal post-transcriptional regulation of iron homeostasis via IRP1 has also been confirmed in previous studies that showed the ferritin mRNA translation repression to be caused by sustained IRP1 activity in PD rodent models as well as patient brains [162,163], and that respiratory dysfunction due to complex I inhibition triggers decreases ISC-biogenesis and induces IRP1 activity [164]. These observations are similar to our data on *Pink1*^−/−^ MEF, as summarized in the Graphical Abstract.

A previous mass spectrometry study in WT rat primary cortical neurons treated with FAC over 24 h showed a 10-fold increase of ferritin protein [54], in good agreement with our findings in WT cells after 24 h. Such induction of ferritin and *Hmox1* has a cytoprotective effect, as previously shown [165,166], but is not maintained in PD brains despite the iron accumulation [99,101]. Inadequate ferritin responses to iron excess would lead to an accumulation of the LIP and mitochondrial iron, a phenomenon that was demonstrated in PD brain tissue by recent studies that employed Mössbauer spectroscopy [167,168]. This scenario is compatible with our proteome findings that MYL6 showed a massive downregulation after FAC selectively in *Pink1*^−/−^ cells, and that PCBP3 exhibited decreased levels without treatment in *Pink1*^−/−^ cells since both factors are involved in iron-chaperone functions to deliver components of the LIP to ferritin [76,169,170].

The abnormally high LIP will bind to the protein Pirin and thus modulate innate immunity via the NFkB pathway (Nuclear Factor “kappa-light-chain-enhancer” of activated B-cells) [109], in good agreement with our observations in the proteome profile that numerous inflammatory factors are dysregulated, and with previous reports that PINK1/PARKIN dysfunction modulates NFkB, causes neuroinflammation, and impairs defenses against invading bacteria [35,38,40,171]. Under iron-saturated conditions, *Aco1*-encoded IRP1 protein functions as aconitase to prevent citrate accumulation in the cytosol, thus harmonizing fatty-acid synthesis and protein acetylation with strong mitochondrial activity, as well as modulating anti-inflammatory responses [172]. It is noteworthy that in *Pink1*^−/−^ flies the neurodegenerative process was shown to depend on the oxidative-stress-triggered inactivation of the labile [4Fe-4S] clusters in the aconitase enzymes [32].

Since iron accumulation is neurotoxic over time [173] and correlates with iron consumption from red meat and bread [174], our observations may also be relevant for dietary approaches that might mitigate disease progression in PD. In the context of dietary prevention of PD, it is important to note that ascorbate (vitamin C) is a modulator of cellular iron import and efflux as well as ferritin biosynthesis and degradation [175,176,177,178,179,180]. Thus, our data may provide a preliminary mechanistic concept to explain the accumulation of iron deposits in the brain of PD patients with PINK1 loss-of-function mutations. In-depth analyses with overexpression and repression of key molecules in brain glia versus neural cells will be necessary to assess this putative scenario. In this context, a recently published study of *Pink1*^−/−^ flies demonstrated increased iron bioavailability in mitochondria via mitoferrin overexpression or ferritin knockdown to be beneficial for motor performance and mitochondrial morphology [181], an observation that contradicts the assumption of iron chelator benefits in PD. The enhanced sensitivity of PINK1-deficient cells to iron overload/toxicity and ferroptosis with reduced lifespan of such organisms might constitute an adverse effect triggered by mechanisms to maximize iron recruitment for inefficient mitochondrial usage.

This study elucidated not only the molecular effects of the PINK1/PARKIN pathway as intrinsic determinants of mitophagy, PD, and longevity. In addition, an important focus of our study was on iron as an extrinsic modulator of these mitochondrial functions, and on the homeostatic expression adaptations that are physiologically induced by iron. Important responses to iron depletion included the slight upregulation of *Abcb10* mRNA contrasted by a converse downregulation of *Slc25a28* (mitoferrin-2), although both transporters are thought to mediate iron import. The substrates of ABCB10 transport activity are currently undefined, but its absence was reported to reduce mitoferrin-1 protein levels, iron import into mitochondria, heme biosynthesis, and hemoglobinization, while a role in the export of ALA (5′-aminolevulinic acid) was excluded [182]. It is difficult to identify the individual substrates for each mitochondrial transporter protein, given that ABCB10, the putative iron-importer mitoferrin, the heme-synthesis factor ferrochelatase, and the ISC-exporter ABCB7 coexist in a protein complex [72,79,83,183] where the deletion of one member may destabilize also its interactors. Similar to ABCB10, a decrease of iron import and heme biosynthesis was also shown upon deletion of mitoferrin [184,185], leading to universal acceptance of mitoferrin as the main mitochondrial iron importer [84]. Upon comparison of the transcriptional regulation of both factors, it is intriguing to note that iron shortage leads to parallel induction of *Tfrc* for iron recruitment across plasma membranes, and induction of *Abcb10* that could recruit iron across mitochondrial membranes, but a converse downregulation of *Slc25a28* which encodes mitoferrin-2. This paradoxical *Slc25a28* downregulation during iron shortage, however, might be expected for a transporter that mediates iron export but is unusual for an import factor. The FAC-triggered upregulation of *Slc25a37*, which encodes mitoferrin-1, at least in *Pink1*^−/−^ cells (Supplementary Figure S5), is expected for a mitochondrial iron import factor, and compatible with the idea of maximized iron recruitment when ISC-biogenesis is impaired. Interestingly, the reduction of *Slc25a28* in *C. elegans* was reported to result in a prolonged lifespan [74]. In only one study so far, ABCB8 was implicated in mitochondrial export functions for iron and factors required for cytosolic ISC-protein maturation [80]. As expected for mitochondrial iron export factors, *Abcb8* expression was downregulated after iron depletion, to a similar level as *Slc25a28*. Regarding the two mitoferrin isoforms, it was also noteworthy that *Slc25a28* responded to iron depletion, whereas *Slc25a37* seemed regulated only after iron excess (Figure S5). Thus, it is also possible that mitoferrin 1 may be better suited to importing iron into mitochondria under conditions of high iron supply (as is the case in erythroid cells in which mitoferrin 1 is the major mitochondrial iron importer). We propose that further studies of transcriptional regulation in response to putative substrate loading would help to elucidate the specific roles of each membrane transporter.

## 4. Materials and Methods

### 4.1. Mouse Embryonic Fibroblast Generation and Culture

The mice used were bred at the Central Animal Facility (ZFE) of the Goethe University Medical Faculty in Frankfurt, under FELASA-certified conditions, in accordance with the ETS123 (European Convention for the Protection of Vertebrate Animals), the Council Directive of 24 November 1986 (86/609/EWG) with Annex II and the German Animal Welfare Act. Approval of the local institutional review board (Regierungspraesidium Darmstadt, project V54-19c20/15-FK/1083) was given on 27 March 2017. MEF were prepared from individual embryos at 14.5 days post-coitus of WT and *Pink1*^−/−^ mice, which were generated and bred as previously reported [20]. In brief, embryos were dissected from the uterus, extremities, and inner organs were removed and the tissue was treated with 0.05% trypsin (Gibco, Thermo Scientific, Schwerte, Germany) for 10–15 min. Cells were cultivated in Dulbecco’s Modified Eagle Medium 4.5 g/L glucose (Invitrogen, Karlsruhe, Germany) plus 15% bovine growth serum (BGS, Thermo Scientific), 1% glutamine, 1% penicillin/streptomycin (all Invitrogen,) at 37 °C and 5% CO_2_ in a humidified incubator, then *Pink1*^−/−^ cells and their respective littermate WT controls were frozen in liquid nitrogen.

### 4.2. Human Fibroblasts

Human fibroblasts of 7 healthy controls and 3 PARK6 patients [17] (passage 8–12) were cultured in Dulbecco’s Modified Eagle Medium 4.5 g/L glucose (Invitrogen) plus 15% bovine growth serum (BGS, Thermo Scientific), 1% glutamine, 1% penicillin/streptomycin (all Invitrogen) at 37 °C and 5% CO_2_ in a humidified incubator.

### 4.3. Iron Overload/Depletion Experiments

MEF or human fibroblast cells were plated in 6-well plates (500,000 cells/well for RT-qPCR and immunoblotting, 1 × 10^6^ for global proteome) and incubated for 24 h. Cells were either left in fresh normal growth medium or treated with 200 µM ferric ammonium citrate (FAC) (Sigma Aldrich, St. Louis, MO, USA), 100 µM 2,2-bipyridyl (22BP) (Roth, Karlsruhe, Germany), or 100 µM Deferoxamine mesylate (DFO) (Sigma Aldrich) in their normal culture medium. Incubation was done for 16 h or 48 h and cell pellets were collected for subsequent RNA and protein isolation, respectively.

### 4.4. Reverse Transcriptase Real-Time Quantitative PCR

For isolation of total RNA, TRI reagent (Sigma Aldrich) was used, and VILO IV (Thermo Scientific) for reverse transcription, both following manufacturers’ instructions. RT-qPCR was performed applying TaqMan Gene Expression Assays (Applied Biosystems, Thermo Scientific) in cDNA from 20 ng total RNA in 20 µL reactions with 2× master mix (Roche, Basel, Switzerland) in a StepOnePlus Real-Time PCR System (Applied Biosystems, Thermo Scientific). An RT-qPCR assay of *Pink1* normalized to *Tbp* was used to confirm the genotype in MEFs. For quantification of the individual mRNA levels, the following TaqMan assays (Thermo Scientific) were employed for mouse: *Abcb6*- Mm00470049_m1, *Abcb7-* Mm01235258_m1, *Abcb8*- Mm00472410_m1, *Abcb10*- Mm00497931_m1, *Abce1*- Mm00649858_m1, *Aco1-* Mm00801417_m1, *Aco2*- Mm00475673_g1, *Alas1-* Mm01235914_m1, *Bach1-* Mm01344527_m1, *Bdh2-* Mm00459075_m1, *Bnip3-* Mm00833810_g1, *Bola1-* Mm01255885_m1, *Brip1*- Mm01297848_m1, *Cdc42bpa*- Mm01322796_m1, *Cisd1*- Mm00728581_s1, *Cisd2*- Mm00835272_m1, *Cp*- Mm00432654_m1, *Ctsb*- Mm01310605_m1, *Ctsd*- Mm00515586_m1, *Ctsf*- Mm00490782_m1, *Cygb* -Mm00446071_m1, *Cyp46a1* -Mm00487306_m1, *Dna2*- Mm01169107_m1, *Dpyd-* Mm00468109_m1, *Egln1*- Mm00459770_m1, *Elp3-* Mm00804536_m1, *Ercc2*- Mm00514776_m1, *Fdx1*- Mm00433246_m1, *Fech*- Mm00500394_m1, *Flvcr1*- Mm01320423_m1, *Foxo3*- Mm01185722_m1, *Fth1-* Mm00850707_m1, *Ftl1-* Mm03030144_g1, *Fxn*- Mm00784016_s1, *Gabarapl1*- Mm00457880_m1, *Glrx5-* Mm00511712_m1, *Hebp1*- Mm00469161_m1, *Hif1a*- Mm00468869_m1, *Hk1*- Mm00439344_m1, *Homer1*- Mm00516275_m1, *Hmox1-* Mm00516005_m1, *Ireb2*- Mm01179595_m1, *Jmjd6*- Mm00466679_m1, *Jund*- Mm04208316_m1, *Keap1*- Mm00497268_m1, *Mef2d-* Mm00504931_m1, *Mitf*- Mm00434954_m1, *Mmp14*- Mm00485054_m1, *Myl6*- Mm02342525_g1, *Ncoa4*- Mm00451095_m1, *Nfe2l2*- Mm00477784_mL, *Nfu1- Mm00777068_m1, Nos2-* Mm_00440502_m1, *Nqo1*- Mm01253561_m1, *Nthl1-* Mm00476559_m1, *P4ha2*- Mm01288628_m1, *Prkn-* Mm00450186_m1, *Pcbp1*- Mm00478712_s1, *Pcbp2*- Mm01296174_g1, *Pcbp3*- Mm01149750_m1, *Pgrmc1*- Mm00443985_m1, *Pink1-* Mm00550827_m1, *Pold1*- Mm00448253_m1, *Ppat*- Mm00549096_m1, *Prdx1*- Mm012619961_s1, *Prim2*- Mm00477104_m1, *Rbfox2*- Mm01197021_m1, *Rsad1*- Mm01296523_m1, *Rsad2*- Mm00491265_m1, *Rrm2*- Mm00485881_m1, *Rtel1*- Mm01220420_m1, *Slc11a2-* Mm00435363_m1, *Slc25a28-* Mm00455077_m1, *Slc25a37*- Mm00471133_m1, *Slc40a1*- Mm01254822_m1, *Sqstm1*- Mm00448091_m1, *Steap2*- Mm01320129_m1, *Steap3*- Mm01287243_m1, *Tbp*- Mm00446973_m1, *Tfeb*- Mm00448968_m1, *Tfrc-*Mm00441941_m1, *Trf- Mm00446715_m1, Tyw5-* Mm01254171_m1. Human TaqMan assays used were: *ABCE1*- Hs01009190_m1, *FOXO3*- Hs00921424_m1, *FTL1*- Hs00830226_gH, *FTH1*- Hs01694011_s1, *HPRT1*- Hs99999909_m1, *KEAP1*- Hs00202227_m1, *RRM2*- Hs00357247_g1, *TFEB*- Hs00292981_m1, *TFRC*- Hs00951083_m1. Results were analyzed with the 2^−ΔΔCT^ method [186].

### 4.5. Quantitative Immunoblotting

Sample preparation for quantitative immunoblotting was done as described before [187]. Samples of 20 μg of protein in 2× Laemmli buffer were heated at 90 °C for 3 min and then separated in 10% tris-glycine polyacrylamide gels, using Precision Plus Protein™ All Blue Standards (Bio-Rad, Hercules, CA, USA) as a size marker. Transfer to nitrocellulose membranes (Protran, GE Healthcare, Thermo Fisher) was done at 50 V over 90 min, with blocking in 5% BSA solution in 1× TBS-T for 1 h at room temperature (RT). Primary antibody incubation against FTH1 (1:1000, Invitrogen, #PA586928), HMOX1 (1:1000, Abcam, Cambridge, UK, ab79854), RRM2 (1:500, Santa Cruz Biotechnolgoy, Santa Cruz, CA, USA, sc-376973), and NCOA4 (1:1000, Santa Cruz sc-373739). Fluorescence-labeled α-rabbit or α-mouse antibodies (1:15,000, Licor Biosciences, Lincoln, NE, USA) were used as secondary antibodies. Normalization occurred with incubation against beta-Actin (1:2000, Sigma Aldrich, A5441). Fluorescence detection occurred on the Licor Odyssey Classic Instrument and bands were densitometrically analyzed with Image Studio Lite, Version 5.2 (Li-Cor Biosciences).

### 4.6. Proteomics Sample Preparation with Label-Free Quantification (LFQ)

Proteomics sample preparation was done according to a published protocol with minor modifications [188]. About 1.5 million cells of *Pink1*^−/−^ MEF and WT cells, either FAC, 22BP, or untreated were lysed in triplicates under denaturing conditions in a buffer containing 3 M guanidinium chloride (GdmCl), 10 mM tris(2-carboxyethyl)phosphine, 40 mM chloroacetamide and 100 mM Tris-HCl pH 8.5. Lysates were denatured at 95 °C for 10 min shaking at 1000 rpm in a thermal shaker and sonicated for 10 min. Lysates were diluted with a dilution buffer containing 10% acetonitrile and 25 mM Tris-HCl, pH 8.0, to reach a 1 M GdmCl concentration. Then, proteins were digested with LysC (Roche; enzyme to protein ratio 1:50, MS-grade) shaking at 700 rpm at 37 °C for 2 h. The digestion mixture was diluted again with the same dilution buffer to reach 0.5 M GdmCl, followed by a tryptic digestion (Roche, enzyme to protein ratio 1:50, MS-grade) and incubation at 37 °C overnight in a thermal shaker at 700 rpm. Peptide desalting was performed according to the manufacturer’s instructions (Pierce C18 Tips, Thermo Scientific, Waltham, MA, USA). Desalted peptides were reconstituted in 1% formic acid in water and half of each sample was further separated into four fractions by strong cation exchange chromatography (SCX, 3M Purification, Meriden, CT, USA). Eluates were first dried in a SpeedVac, then dissolved in 5% acetonitrile and 2% formic acid in water, briefly vortexed, and sonicated in a water bath for 30 s prior injection to nano-LC-MS.

### 4.7. LC-MS/MS Instrument Settings for Shotgun Proteome Profiling and Data Analysis

LC-MS/MS was carried out by nanoflow reverse phase liquid chromatography (Dionex Ultimate 3000, Thermo Scientific) coupled online to a Q-Exactive HF Orbitrap mass spectrometer (Thermo Scientific), as reported previously [189]. Briefly, the LC separation was performed using a PicoFrit analytical column (75 μm ID × 50 cm long, 15 µm Tip ID; New Objectives, Woburn, MA, USA) in-house packed with 3-µm C18 resin (Reprosil-AQ Pur, Dr. Maisch, Ammerbuch, Germany). Peptides were eluted using a gradient from 3.8% to 38% solvent B in solvent A over 120 min at 266 nL per minute flow rate. Solvent A was 0.1% formic acid and solvent B was 79.9% acetonitrile, 20% H_2_O, 0.1% formic acid. For the IP samples, a one hour gradient was used. Nanoelectrospray was generated by applying 3.5 kV. A cycle of one full Fourier transformation scan mass spectrum (300−1750 *m*/*z*, resolution of 60,000 at *m*/*z* 200, automatic gain control (AGC) target 1 × 10^6^) was followed by 12 data-dependent MS/MS scans (resolution of 30,000, AGC target 5 × 10^5^) with a normalized collision energy of 25 eV. In order to avoid repeated sequencing of the same peptides, a dynamic exclusion window of 30 s was used. In addition, only peptide charge states between two to eight were sequenced.

Raw MS data were processed with MaxQuant software (v1.6.10.43, from Max Planck Institute of Biochemistry, Martinsried, Germany) and searched against the mouse proteome database UniProtKB with 55,153 entries, released in August 2019. Parameters of MaxQuant database searching were a false discovery rate (FDR) of 0.01 for proteins and peptides, a minimum peptide length of seven amino acids, a first search mass tolerance for peptides of 20 ppm and a main search tolerance of 4.5 ppm, and using the function “match between runs”. A maximum of two missed cleavages was allowed for the tryptic digest. Cysteine carbamidomethylation was set as fixed modification, while N-terminal acetylation and methionine oxidation were set as variable modifications. The correlation analysis of biological replicates and the calculation of significantly different proteins were done with Perseus (v1.6.10.43, from Max Planck Institute of Biochemistry). LFQ intensities, originating from at least two different peptides per protein group were transformed by log_2_. Only groups with valid values in at least one group were used, missing values were replaced by values from the normal distribution. Statistical analysis was done by a two-sample t-test with Benjamini–Hochberg (BH, FDR of 0.05) correction for multiple testing. Significantly regulated proteins between the conditions were indicated by a plus sign in Supplementary Table S1.

### 4.8. Statistical Evaluation

RT-qPCR and quantitative immunoblot result were analyzed using Graphpad Prism Version 8 (GraphPad, San Diego, CA, USA) and significant differences were calculated with two-way ANOVA with subsequent multiple comparison tests.

### 4.9. Visualization

Graphs were created using Graphpad Prism Version 8; Tables were assembled using Microsoft Excel, and Venn diagrams were created with the online tool for Venn diagrams of Bioinformatics & Evolutionary Genomics (http://bioinformatics.psb.ugent.be/webtools/Venn/, accessed on 18.09.2020)

## 5. Conclusions

We conducted a pioneering survey of global proteomic adaptations and transcriptional responses to extracellular iron changes, using murine fibroblasts from WT and *Pink1*^−/−^ animals, to then validate prominent findings in fibroblasts from *PARK6* patients. The proteome profile identified 4 novel factors to be conversely regulated upon iron deficiency versus iron excess, namely CPT1A, MMP14, XDH, and PYGL. The transcriptional regulation profile argued against previous notions that SLC25A28 has mitochondrial import function, providing evidence that SLC25A28 expression is downregulated by iron shortage, while SLC25A37 responds to iron excess.

Iron deprivation had massive effects on proteome and transcript levels and they are relevant when trying long-term neuroprotective therapy of PD patients by iron chelators. On the one hand, iron shortage upregulated the alpha-synuclein antagonist SNCB at the protein level, and *Pink1* and *Prkn* at the mRNA level, possibly ensuring neuroprotection and chronic maximized mitophagy, which might have beneficial effects for the aggregation and mitochondrial dysfunction in PD tissue. On the other hand, iron shortage decreased the abundance of iron-binding nucleotide synthesis factors such as RRM2, of ISC biogenesis factors, and ISC-associated guardians of RNA/DNA stability, limiting also the transcriptional expression e.g., for *Ppat, Nthl1*, *Dna2*, *Pold1*, *Tyw5,* and *Prim2*, so the known vulnerability of the mitochondrial genome in PD might be enhanced in deleterious fashion by iron chelators. Particularly the impairment of DNA repair is known as a risk factor of neurodegenerative diseases and a modulator of lifespan [13,14,15]. Largely, the factors identified act via iron homeostasis and mitophagy to alter the health period [11,12].

Even at untreated conditions, the *Pink1*-ablation mediated a deficit of the LIP chaperone PCBP3 protein, the ISC-biogenesis factor GLRX5 protein, and the ISC-associated *Abce1* transcript as a growth-limiting effect. These data provide the first evidence in mammals for the existence of a PINK1-dependent mechanism described previously in *D. melanogaster* [122,124,148], where nucleus-encoded mRNAs and the corresponding precursor proteins for mitochondria undergo co-translational quality control by ABCE1, in parallel to the mitophagic elimination of dysfunctional mitochondrial fragments.

As a prominent finding after iron overload, the transcriptional ferritin induction was potentiated in *Pink1*-ablated cells, while the ferritin protein levels were diminished, a contrast that might be due to post-transcriptional translation repression caused by reduced ISC-availability/stability.

Taken together, this preliminary documentation of on-demand regulations in fibroblasts should be complemented by studies of neural cells, mitochondrial fractions, long-term effects, and overexpression/depletion of crucial factors, to confirm the mechanisms and the relevance for PD. The data point to inefficient iron usage in the mitochondria of PINK1-mutant cells, followed by maximized iron import and mobilization to ensure sufficient DNA/RNA quality, via ISC-associated enzymes. This observation clarifies in what pathways an iron chelator therapy of PD may have long-term adverse effects and explains why acutely improved motor performance can be observed in PINK1-mutant flies after iron supplementation.

## Figures and Tables

**Table 1:**
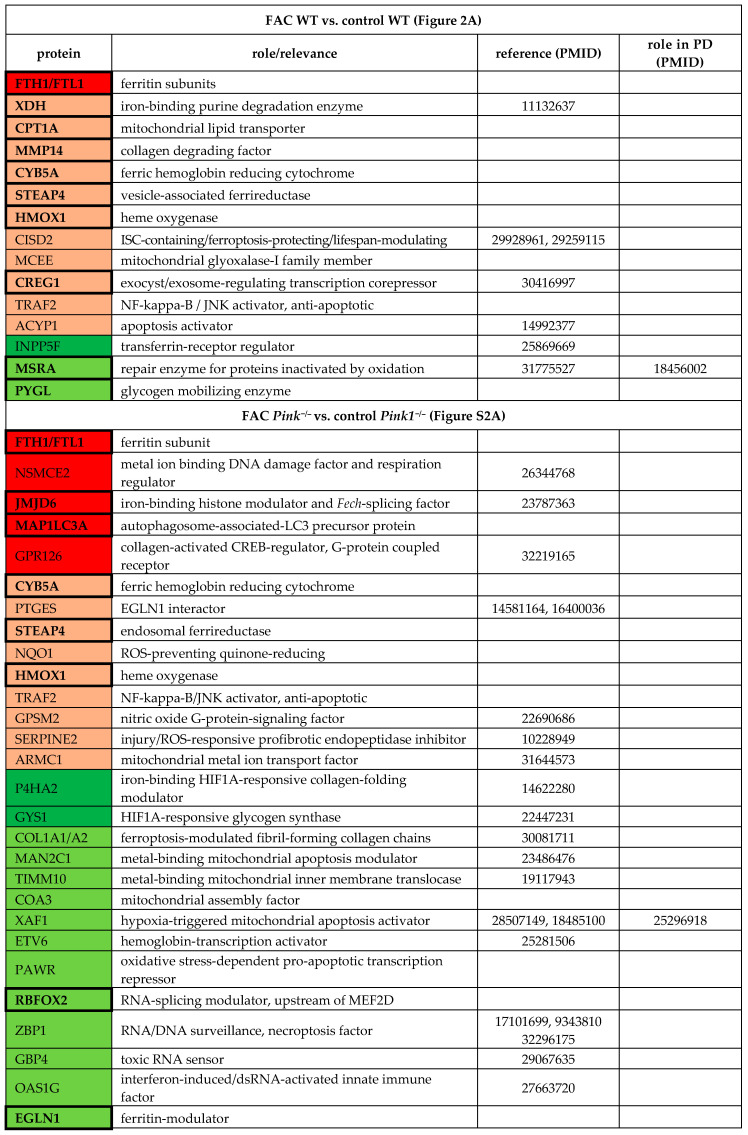
FAC effects. Overview of selected significantly changed factors in the global proteome of MEF, comparing FAC WT vs. control WT, and FAC *Pink1*^−/−^ vs. control *Pink1*^−/−^, listing their respective roles and literature references (PubMedIDs), and highlighting special relevance for Parkinson’s disease (PD). Upregulations are shown with dark red (FDR > 0.05) and light red (*p* > 0.05), whereas downregulations are marked with dark green (FDR > 0.05) and light green (*p* > 0.05). Factors that show converse regulation after iron overload versus iron depletion and thus appear in several tables are marked with bold fonts. Factors with dysregulation in diverse conditions are emphasized by increased table cell border thickness. For details, see Figure 2 and Supplementary Table S1.

**Table 2:**
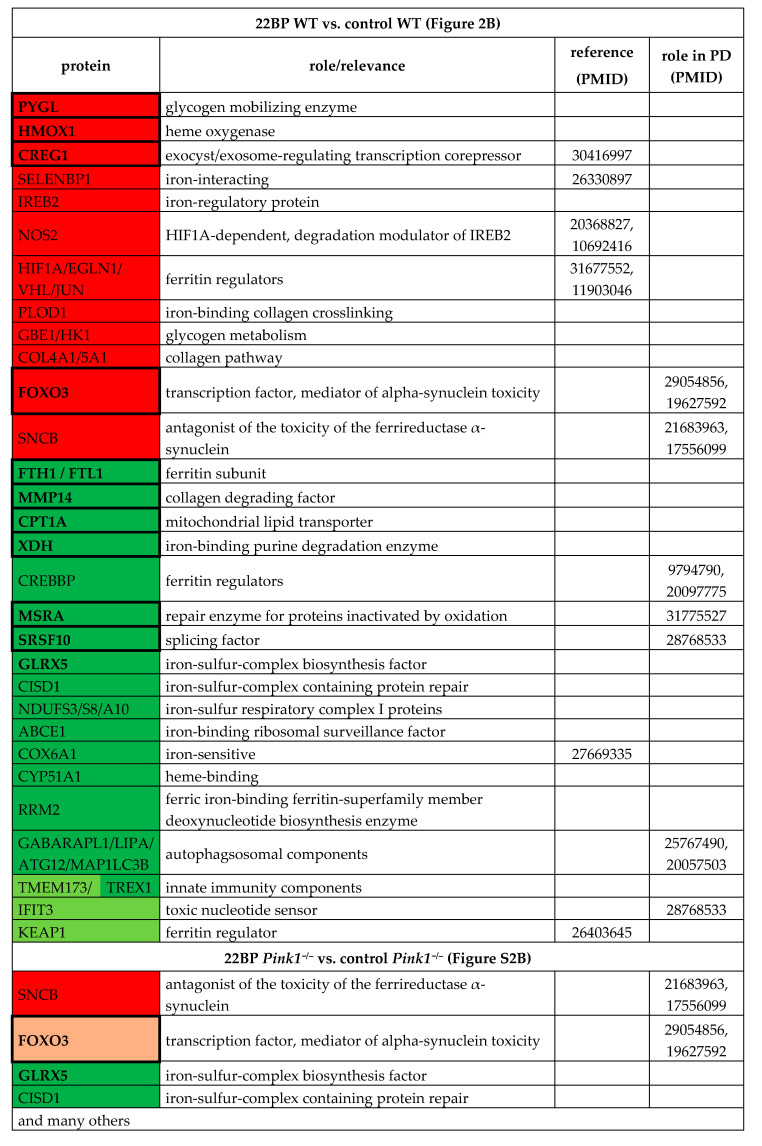
22BP effects. Overview of selected significantly changed factors in the global proteome of MEF, comparing 22BP WT vs. control WT, and 22BP *Pink1*^−/−^ vs. control *Pink1*^−/−^, listing their respective roles and PMIDs, and highlighting special relevance for PD. Upregulations are shown with dark red (FDR > 0.05) and light red (*p* > 0.05), whereas downregulations are marked with dark green (FDR > 0.05) and light green (*p* > 0.05). Factors that show converse regulations after iron overload versus iron depletion and thus appear in several tables are marked with bold fonts. Factors with dysregulations in diverse conditions are emphasized by increased table cell border thickness. For details, see Figure 2 and Supplementary Table S1.

**Table 3:**
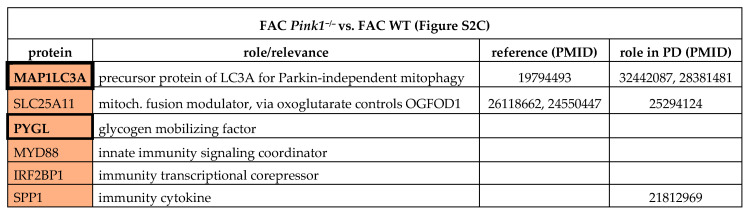

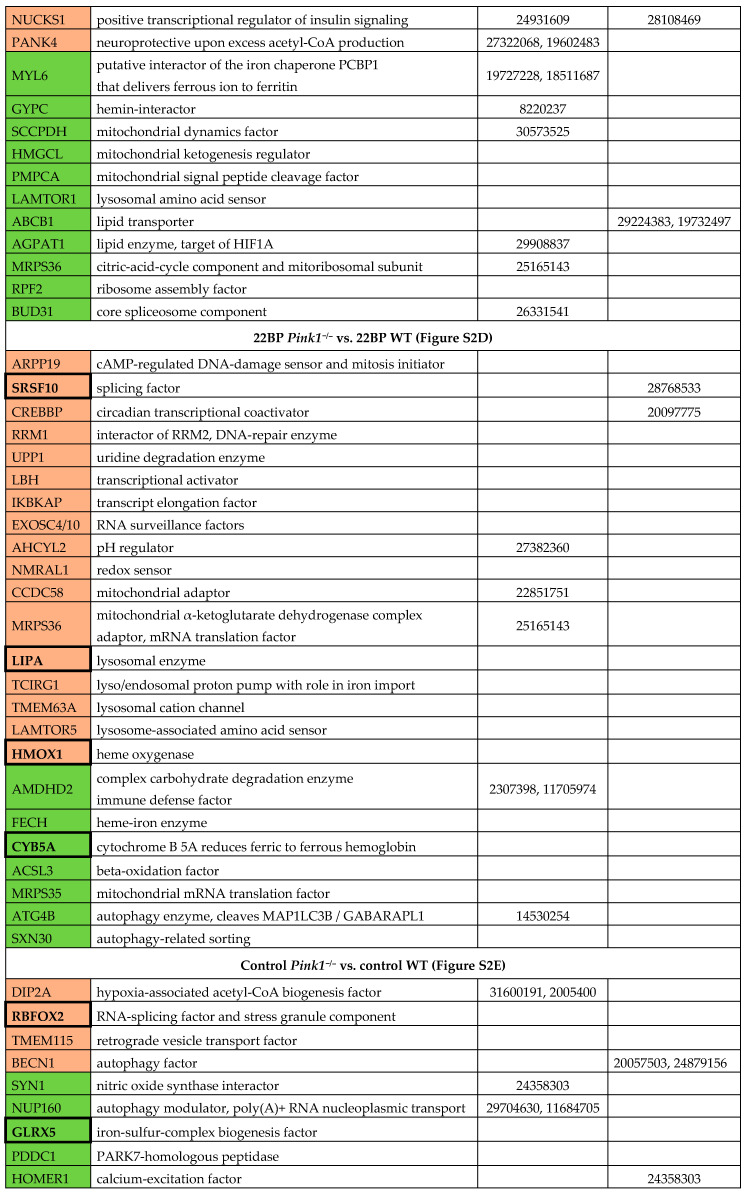

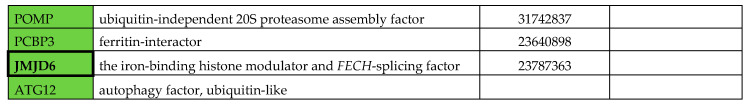
PINK1 effects. Overview of selected significantly changed factors in the global proteome of MEF, comparing FAC *Pink1*^−/−^ vs. FAC WT, 22BP *Pink1*^−/−^ vs. 22BP WT, and control *Pink1*^−/−^ vs. control WT, listing their respective roles and PMIDs, and highlighting special relevance for PD. Upregulations are shown with dark red (FDR > 0.05) and light red (*p* > 0.05), whereas downregulations are marked with dark green (FDR > 0.05) and light green (*p* > 0.05). Factors that show converse regulations after iron overload versus iron depletion and thus appear in several tables are marked with bold fonts. Factors with dysregulations in diverse conditions are emphasized by increased table cell border thickness. For details, see Figure 2 and Supplementary Table S1.

## Supplementary Materials

The following are available online at https://www.mdpi.com/2073-4409/9/10/2229/s1. Table S1, Perseus software output of global proteome analyses; Table S2, Statisctical details of transcriptional changes; Figure S1, Dysregulations in global proteome of WT MEF after FAC-treatment; Figure S2, Volcano plots of global proteome profiles; Figure S3, STRING diagram of consistently dysregulated factors; Figure S4, Dysregulations in global proteome of WT MEF after 22BP-treatment; Figure S5, Transcriptional dysregulations upon RT-qPCR analyses; Figure S6, Transcriptional dysregulations of antioxidant and hypoxic control.

## Abbreviations

µM	MicroMolar
22BP	2,2′-Bipyridine
Abcb1	ATP binding cassette subfamily B member 1
Abcb7	ATP binding cassette subfamily B member 7
Abcb8	ATP binding cassette subfamily B member 8
Abcb10	ATP binding cassette subfamily B member 10
Abce1	ATP binding cassette subfamily E member 1, RNase L inhibitor 1
Aco1	Aconitase 1, cytoplasmic, aka Irp1, aka Ferritin repressor protein
Aco2	Aconitase 2, mitochondrial
ACSL3	Acyl-CoA synthetase long-chain family member 3
ACTB	Actin beta
ACYP1	Acylphosphatase 1
AGPAT1	1-Acylglycerol-3-Phosphate O-Acyltransferase 1
AHCYL2	Adenosylhomocysteinase like 2
aka	also known as
ALA	5′-aminolevulinic acid
Alas1	5′-aminolevulinate synthase 1
AMDHD2	Amidohydrolase domain containing 2
ANOVA	Analysis of variance
ARMC1	Armadillo repeat containing 1
ARPP19	CAMP regulated phosphoprotein 19
ARNT	Aryl hydrocarbon receptor nuclear translocator
ATG4B	Autophagy related 4B cysteine peptidase
ATG12	Autophagy related 12
ATPase	Adenosintriphosphatase
Bdh2	3-hydroxybutyrate dehydrogenase-2
BECN1	Beclin 1, Autophagy related
Bola1	BolA Family Member 1
Brip1	Brca1 Interacting Protein C-Terminal Helicase 1, Fancj
BUD31	BUD31 homolog, Functional spliceosome-associated protein 17
cAMP	Cyclic adenosine monophosphate
CCDC58	Coiled-coil domain containing 58
Cdc42bpa	Cdc42 binding protein kinase alpha (Dmpk-like), Mrcka
CIA	Cytosolic iron-sulfur cluster assembly machinery
Cisd1	CDGSH iron-sulfur domain 1
Cisd2	CDGSH iron-sulfur domain 2, Wfs2
CO_2_	Carbon dioxide
COA3	Cytochrome C oxidase assembly factor 3
COL1A1/2	Collagen type I alpha 1 chain, Collagen type I alpha 2 chain
COL4A1	Collagen type IV alpha 1 chain
COL5A1	Collagen type IV alpha 1 chain
COX1	Mitochondrially encoded cytochrome C oxidase I
COX6A1	Cytochrome C oxidase subunit 6A1, mitochondrial
Cp	Ceruloplasmin
CPT1A	Carnitine palmitoyltransferase 1A
CREBBP	CREB binding protein
CREG1	Cellular repressor of E1A stimulated genes 1
CYB5A	Cytochrome B5 type A
Cyp46a1	Cytochrome P450 Family 46 Subfamily A Member 1
Cyp56a1	Cytochrome P450 Family 51 Subfamily A Member 1
DFO	Deferoxamine
DIP2A	Disco interacting protein 2 homolog A
Dmt1	Divalent metal transporter 1, encoded by *Slc11a2*
DNA	Desoxyribonucleic acid
Dna2	DNA replication helicase/nuclease 2
Dpyd	Dihydropyrimidine dehydrogenase
Elp3	Elongator acetyltransferase complex subunit 3
EGLN1	Egl-9 family hypoxia-inducible factor 1
ER	Endoplasmic reticulum
Ercc2	ERCC excision repair 2, TFIIH core complex helicase subunit, Cxpd
ETV6	ETS variant transcription factor 6
EXOSC4/10	Exosome component 4/10
FAC	Ferric ammonium acid
Fdx1	Ferredoxin 1, mitochondrial Adrenodoxin
Fech	Ferrochelatase, Heme synthase
Flvcr1a	Feline leukemia virus subgroup C cellular receptor 1a, Pcarp
Flvcr1b	Feline leukemia virus subgroup C cellular receptor 1b, Pcarp
FOXO3	Forkhead box O3
Fth1	Ferritin heavy chain
Ftl1	Ferritin light chain
Ftmt	Mitochondrial ferritin
Fxn	Frataxin, aka Frda
g	Gram
GABARAPL1	GABA type A receptor associated protein like 1
GBE1	Glucan (1,4-Alpha-), branching enzyme 1
GBP4	Guanylate binding protein 4
Glrx5	Glutaredoxin 5
GPR126	G-Protein coupled receptor 126
GPSM2	G Protein signaling modulator 2
GYPC	Glycophorin C
GYS1	Glycogen synthase 1
h	Hour
Hbs1l	Hbs1 like translational GTPase, Erfs
Hebp1	Heme binding protein 1
HIF1A	Hypoxia-Inducible Factor 1-alpha
HK1	Hexokinase 1
HMGCL	Mitochondrial 3-Hydroxy-3-Methylglutaryl-CoA Lyase
Hmox1	Heme oxygenase 1, HO-1
HOMER1	Homer scaffold protein 1
IFIT3	Interferon induced protein with tetratricopeptide repeats 3
IKBKAP	IkappaB kinase complex-associated protein, Elp1
IMPC	International mouse phenotyping consortium
INPP5F	Inositol Polyphosphate-5-Phosphatase F
IRE	Iron response element
Ireb1	Iron-responsive element-binding protein 1 (Irp1), encoded by *Aco1*
Ireb2	Iron-responsive element-binding protein 2 (Irp2), encoded by *Ireb2*
IRF2BP1	Interferon regulatory factor 2 binding protein 1
Irp1	Iron regulatory protein 1 (cytosolic aconitase = Ireb1), encoded by *Aco1*
Irp2	Iron regulatory protein 2 (Aco3 = Ireb2), encoded by *Ireb2* mRNA
ISC	Iron-sulfur-cluster
JMJD6	Jumonji domain protein 6, Arg-demethylase, Lys-hydroxylase
JUN	Jun proto-oncogene, AP-1 transcription factor subunit
KEAP1	Kelch-like ECH associated protein 1
L	Liter
LAMTOR1/5	Late endosomal/lysosomal adaptor, MAPK and MTOR activator 1/5
LBH	LBH regulator of WNT signaling pathway
LFQ	Label free quantification
LIP	Labile iron pool
LIPA	Lipase A, lysosomal acid type
MAN2C1	Mannosidase alpha class 2C member 1
MAP1LC3A/B	Microtubule associated protein 1 light chain 3 alpha/beta
MCEE	Methylmalonyl-CoA Epimerase, mitochondrial
MEF	Mouse embryonic fibroblast
MEF2D	Myocyte enhancer factor 2D
Mfrn1	Mitoferrin 1, encoded by *Slc25a37*
Mfrn2	Mitoferrin 2, encoded by *Slc25a28*
Min	Minute
MMP14	Matrix metallopeptidase 14
MRPS36	Mitochondrial ribosomal protein S36
MSRA	Methionine sulfoxide reductase A
mRNA	Messenger ribonucleic acid
mTOR	Mechanistic target of rapamycin kinase
MPTP	1-methyl-4-phenyl-1,2,3,6-tetrahydropyridine
MYD88	Myeloid differentiation primary response protein MyD88
MYL6	Myosin light chain 6
Ncoa4	Nuclear receptor coactivator 4
NDUFA10	NADH:Ubiquinone oxidoreductase subunit A10
NDUFS3/8	NADH:Ubiquinone oxidoreductase core subunit S3/subunit S8
NFkB	Nuclear Factor ‘kappa-light-chain-enhancer’ of activated B-cells
Nfu1	Nfu1 iron-sulfur cluster scaffold
NMRAL1	NmrA like redox sensor 1
NOS2	Nitric oxide synthase 2
Not4	Ccr4-Not transcription complex subunit 4
NQO1	NAD(P)H quinone dehydrogenase 1
NRF2	Nuclear factor, erythroid 2 like 2, Nfe2l2
NSMCE2	Non-structural maintenance of chromosomes element 2 homolog
Nthl1	Nth like DNA glycosylase 1
NUCKS1	Nuclear casein kinase and cyclin-dependent kinase substrate 1
NUP160	Nucleoporin 160
OAS1G	2′-5′-Oligoadenylate synthetase 1
P4HA2	Prolyl 4-hydroxylase subunit alpha 2
PANK4	Pantothenate kinase 4
Parkin	Parkinson disease protein 2
PAWR	Pro-apoptotic WT1 regulator
Pcbp1	Poly(RC) binding protein 1, hnRNP-E1
Pcbp2	Poly(RC) binding protein 2, hnRNP-E2
PD	Parkinson’s disease
PDDC1	Glutamine amidotransferase like Class 1 domain containing 1, Gatd1
Pgrmc1	Progesterone receptor membrane component 1, Dap1
Pink1	PTEN induced kinase 1
PLOD1	Procollagen-lysine,2-oxoglutarate 5-dioxygenase 1
PMPCA	Peptidase, mitochondrial processing subunit alpha
Pold1	DNA polymerase delta 1 catalytic subunit, Cdc2 homolog
POMP	Proteasome maturation protein
Ppat	Phosphoribosyl pyrophosphate amidotransferase, Gpat, ATase
Prim2	DNA primase subunit 2
PTGES	Prostaglandin E synthase
PMID	PubMed database of medical literature, reference IDentifier number
PYGL	Glycogen Phosphorylase L
RBFOX2	RNA binding fox-1 homolog 2
RNA	Ribonucleic acid
ROS	Reactive oxygen species
RPF2	Ribosome production factor 2 homolog
RRM1/2	Ribonucleotide reductase regulatory subunit M1/M2
rRNA	Ribosomal RNA
Rsad1	Radical S-adenosyl methionine domain containing 1
Rsad2	Radical S-adenosyl methionine domain containing 2
RT	Room temperature
RT-qPCR	reverse-transcriptase real-time quantitative polymerase chain reaction
Rtel1	Regulator of telomere elongation helicase 1
SCCPDH	Saccharopine dehydrogenase
SEM	Standard error of the mean
SELENBP1	Selenium binding protein 1
SERPINE2	Serpin family E member 2
SH2B1	SH2 domain-containing protein 1B
Slc11a2	Solute carrier family 11 member 2, Divalent metal transporter 1, Dmt1
Slc25a37	Solute carrier family 25 member 37, Mitoferrin 1
Slc25a28	Solute carrier family 25 member 28, Mitoferrin 2
Slc40a1	Solute carrier family 40 member 1, Ferroportin 1, Ireg1
SNCB	Synuclein beta
SMX30	Sorting nexin family member 30, ATG24A
SPP1	Secreted phosphoprotein 1
SRSF10	Serine and arginine-rich splicing factor 10
SSBP1	Single-stranded DNA binding protein 1
Steap2	Six transmembrane epithelial antigen of the prostate 2, Stmp
Steap3	Six transmembrane epithelial antigen of the prostate 3, Stmp3
Steap4	Six transmembrane epithelial antigen of the prostate 4, Stamp2
STRING	Search Tool for the Retrieval of Interacting Genes/Proteins
SYN1	Synapsin I
Tbp	Tata-binding protein
TBST	Tris-buffered saline/Tween 20
TCA	Tricarboxylic acid cycle
TCIRG1	T Cell immune regulator 1, ATPase H+ transporting V0 subunit A3
TIMM10	Translocase of inner mitochondrial membrane 10
TMEM115	Transmembrane protein 115
TMEM173	Stimulator of interferon genes protein, Sting
TMEM63A	Transmembrane protein 63A
TRAF2	TNF receptor-associated factor 2
TREX1	Three prime repair exonuclease 1
Trf	Transferrin
Tfrc	Transferrin receptor 1
Tyw5	tRNA wybutosine synthesizing protein 5
UPP1	Uridine phosphorylase 1
UTR	Untranslated region
V	Volt
VHL	Von Hippel-Lindau tumor suppressor
WT	Wildtype
XAF1	XIAP associated factor 1
XDH	Xanthine dehydrogenase
ZBP1	Z-DNA binding protein 1
