# Supplementary figures and images for: Systematic Surveys of Iron Homeostasis Mechanisms Reveal Ferritin Superfamily and Nucleotide Surveillance Regulation to be Modified by PINK1 Absence
Source: Cells. 2020 Oct 2;9(10):2229. doi: 10.3390/cells9102229 (PMC7650593; doi:10.3390/cells9102229)

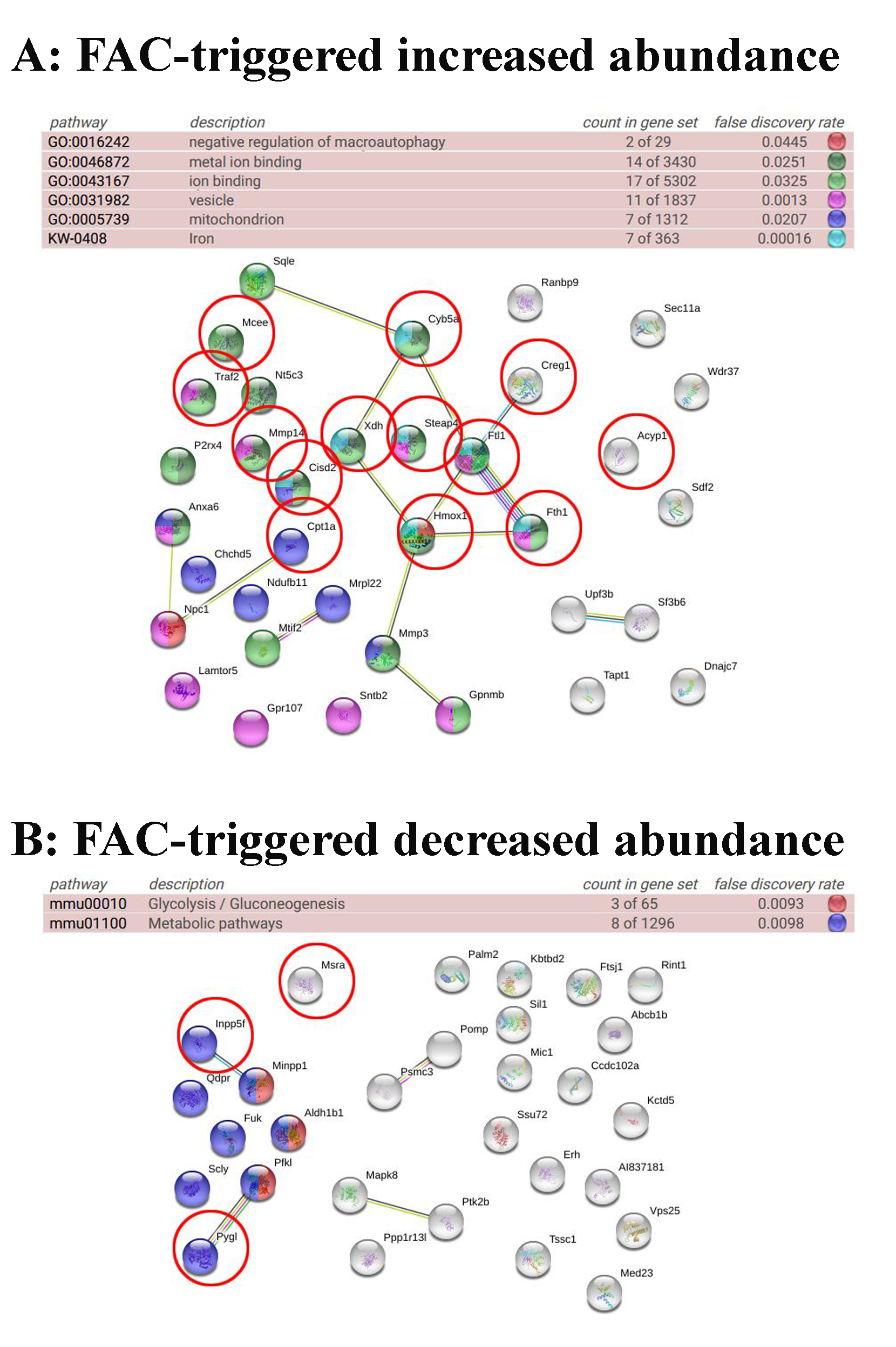

Supplement: Supplementary file 1 [file cells-09-02229-s001.zip › KeyAuburger_iron_longevity_SupplementaryFigure_S1_flat.tif]

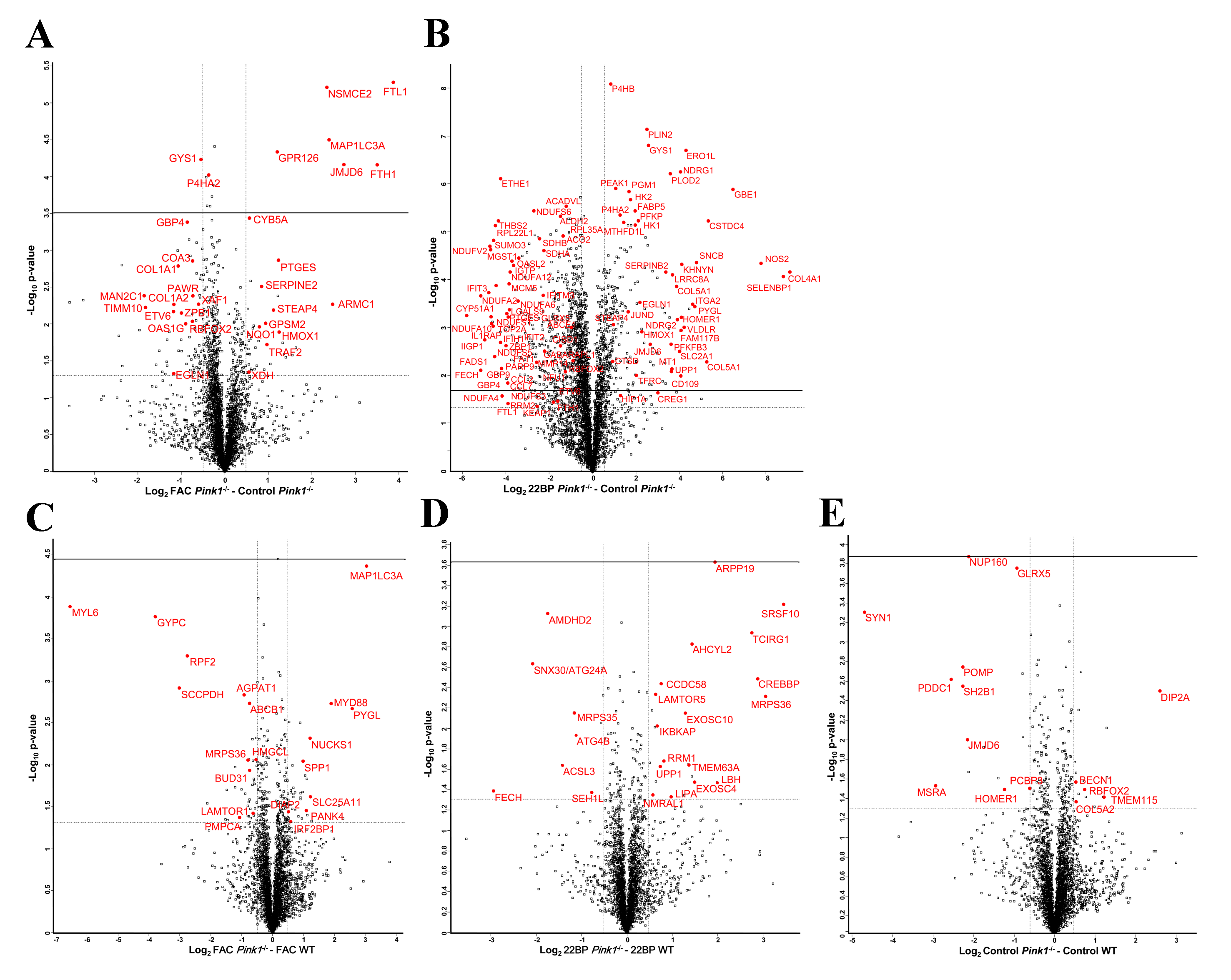

Supplement: Supplementary file 1 [file cells-09-02229-s001.zip › KeyAuburger_iron_longevity_SupplementaryFigure_S2_new_flat.tif]

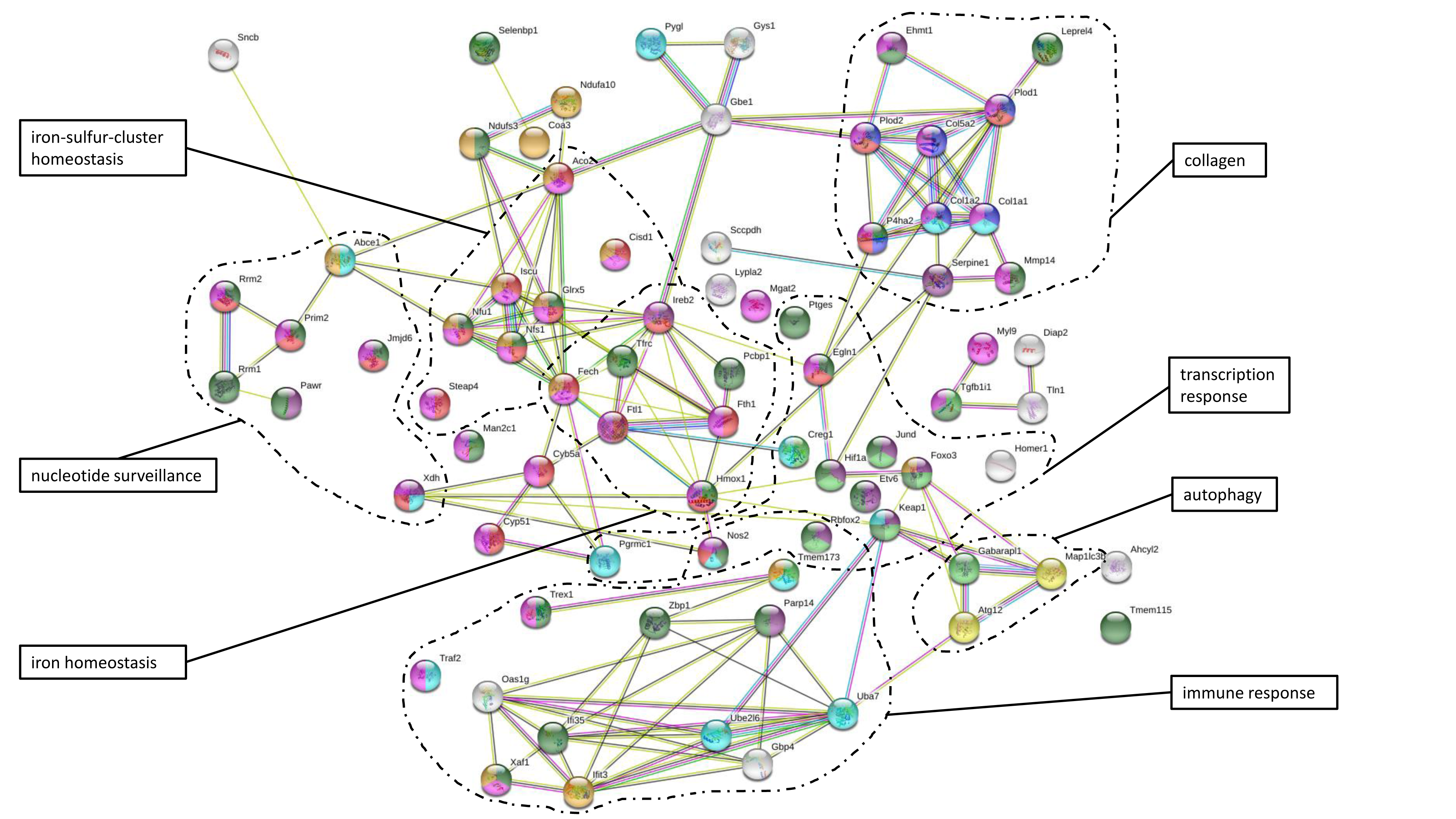

Supplement: Supplementary file 1 [file cells-09-02229-s001.zip › KeyAuburger_iron_longevity_SupplementaryFigure_S3_flat.tif]

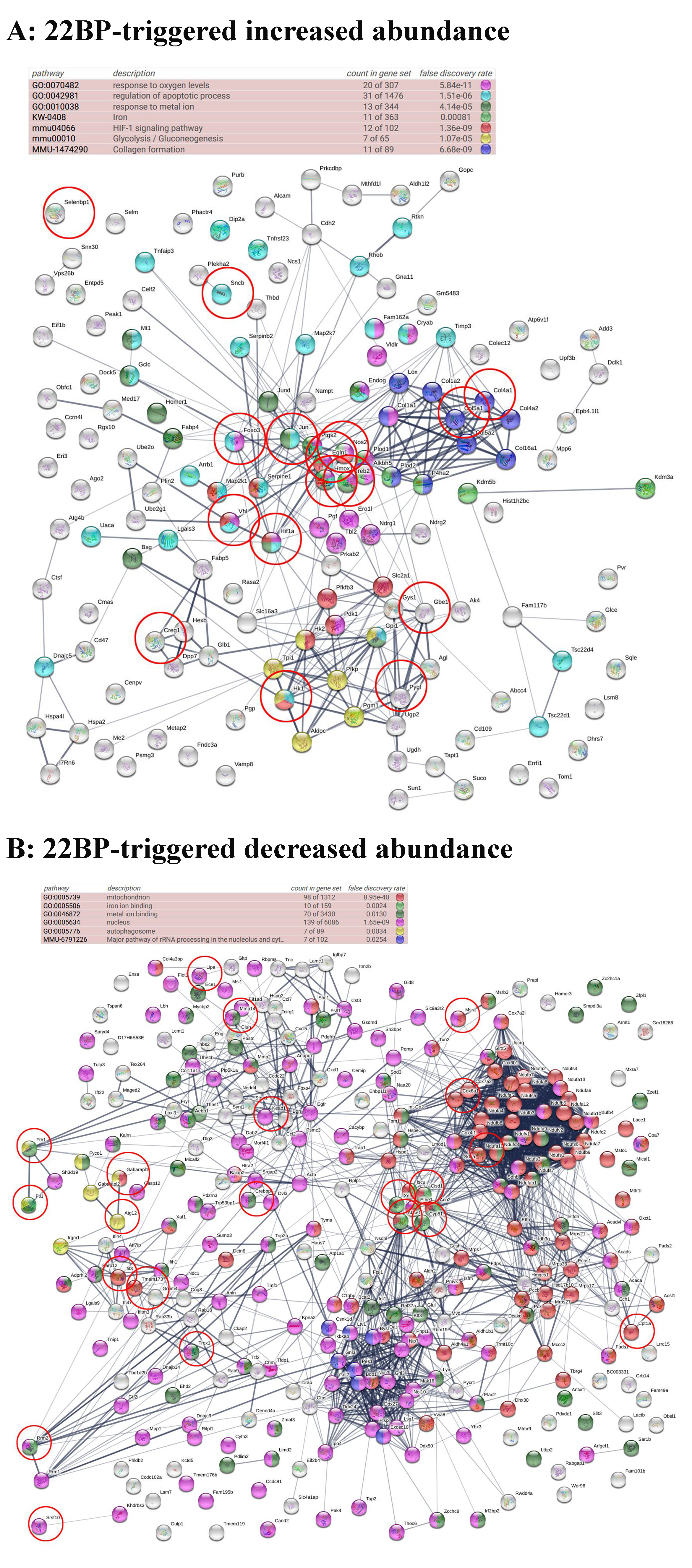

Supplement: Supplementary file 1 [file cells-09-02229-s001.zip › KeyAuburger_iron_longevity_SupplementaryFigure_S4_flat.tif]

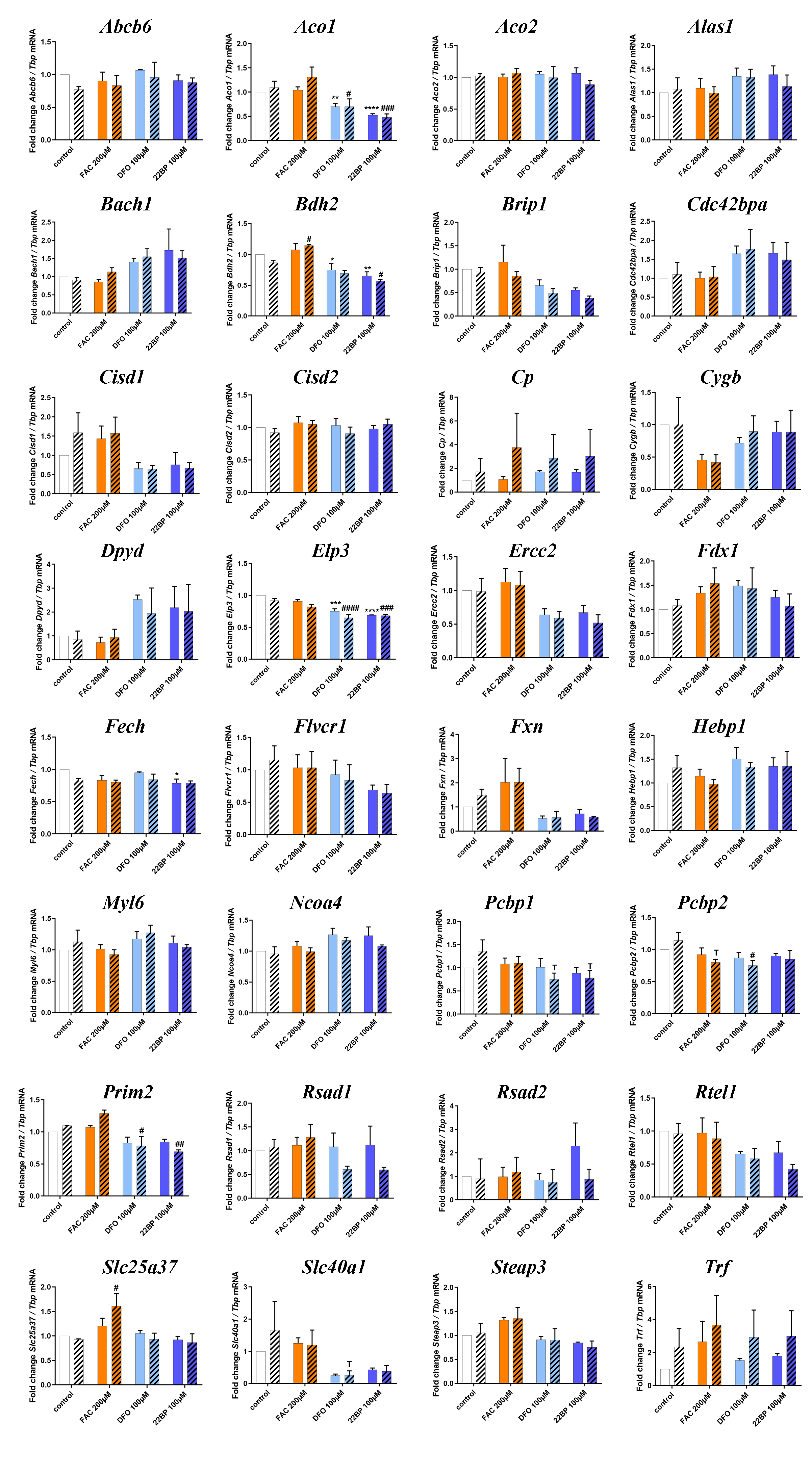

Supplement: Supplementary file 1 [file cells-09-02229-s001.zip › KeyAuburger_iron_longevity_SupplementaryFigure_S5_new_flat.tif]

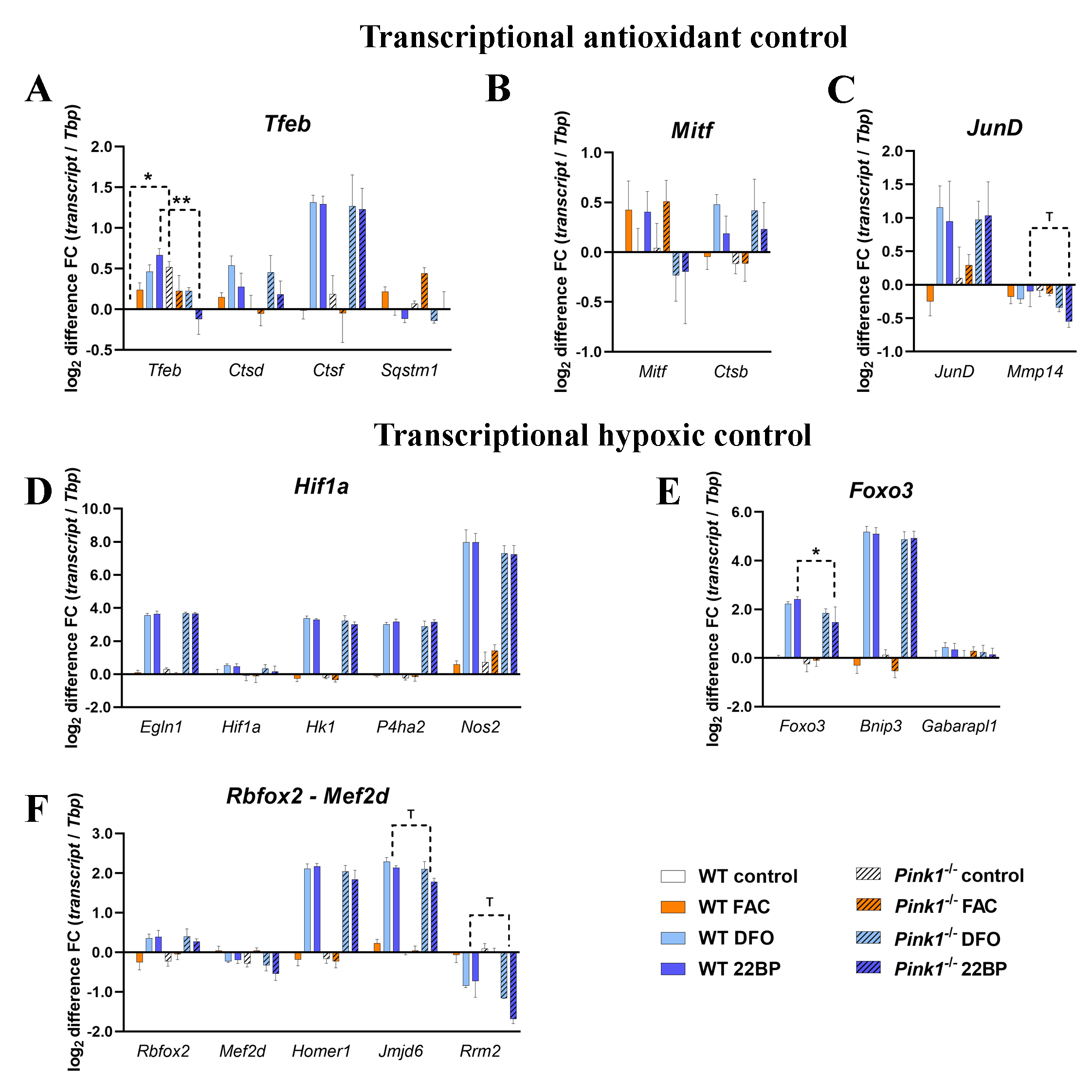

Supplement: Supplementary file 1 [file cells-09-02229-s001.zip › KeyAuburger_iron_longevity_SupplementaryFigure_S6_new_flat.tif]
